# Targeting macrophage metabolism: mechanisms, cellular crosstalk and implications in obesity-associated metabolic diseases

**DOI:** 10.3389/fimmu.2026.1819389

**Published:** 2026-07-10

**Authors:** Chensi Yao, Wei Zheng, Yamei Jin, Jiahao Sheng, Mengjiao Kang, Jijuan Zhong, Min Li, Liyun Duan, Xiao Yuan

**Affiliations:** 1 Department of Endocrinology, The First Affiliated Hospital of Zhejiang Chinese Medical University (Zhejiang Provincial Hospital of Chinese Medicine), Hangzhou, China; 2 The First Clinical Medical School, Zhejiang Chinese Medical University, Hangzhou, China; 3 Molecular Biology Laboratory, Guang’anmen Hospital, China Academy of Chinese Medical Sciences, Beijing, China; 4 The First Clinical Medical College, Shandong University of Traditional Chinese Medicine, Jinan, China; 5 Affiliated Hospital of Shandong University of Traditional Chinese Medicine, Jinan, China

**Keywords:** inflammation, macrophages, metabolism, obesity-associated metabolic diseases, polarization, reprogramming

## Abstract

Obesity and its associated metabolic disorders constitute a prominent public health challenge. Diverse environmental and metabolic cues trigger alterations in macrophage metabolism, thereby influencing their functional phenotypes. Due to their phenotypic plasticity, macrophages play beneficial roles in tissue homeostasis, yet they also contribute to the progression of metabolic diseases. Consequently, beyond systemic chronic low-grade inflammation, greater attention should be directed toward immunometabolic dysfunction in metabolic tissues during obesity and its related diseases. This review summarizes the functional phenotypes and metabolic characteristics of macrophages, with an emphasis on how tissue niches influence macrophage function in the context of various obesity-related metabolic diseases. Enhanced understanding of the interplay between macrophages and metabolic target organs/tissues may provide novel therapeutic strategies for managing obesity and associated metabolic disorders.

## 1 Introduction

Obesity is a chronic metabolic disorder resulting from an imbalance between energy intake and expenditure, characterized by excessive accumulation of adipose tissue, which often leads to a low-grade, persistent, systemic sterile inflammatory state (1). According to World Health Organization data, over 1 billion people worldwide are currently classified as having obesity, with statistics across all regions showing alarming increases in prevalence rates among all age groups (2), making it a growing global health concern. Mounting evidence indicates that obesity serves as a significant risk factor for multiple metabolic disorders, including insulin resistance (IR), type 2 diabetes (T2D), non-alcoholic fatty liver disease (NAFLD), dyslipidemia, and cardiovascular diseases (CVD) (3). Over the past two decades, mortality rates from metabolic diseases have steadily increased, with obesity accounting for approximately 40% of these deaths and causing 5 million fatalities in 2019 alone (4).

Current research reveals that disruption of immune-metabolic homeostasis is a hallmark of obesity-related metabolic diseases, where chronic low-grade inflammation is driven not only by immune cell functional impairment, but also by profound phenotypic switching and altered subset distribution of immune cells, alongside excessive production of pro-inflammatory cytokines. This intimate interplay between immunity and metabolism is conceptualized as “immunometabolism” —defined as the changes in intracellular metabolic pathways in immune cells during activation (5). This concept encompasses two interrelated dimensions. At the cellular level, dynamic reprogramming of core metabolic pathways—including glycolysis, the tricarboxylic acid cycle, and lipid and amino acid metabolism—directs immune cell differentiation, polarization, and effector functions. At the systemic level, activated immune cells in turn exert reciprocal effects on whole-body metabolic homeostasis. This bidirectional interplay between immune cells and systemic metabolism has emerged as a central paradigm for understanding obesity-associated metabolic disorders.

As the most crucial innate immune cells, macrophages play a pivotal role in maintaining the balance between homeostasis and disease, including the development of tissues and organs, defense against pathogen invasion, inflammatory responses, and tissue repair. Macrophage infiltration constitutes a primary cause of obesity-related metabolic diseases. Recent research has garnered significant attention regarding the role of macrophage fate and function in regulating and sustaining metabolic homeostasis. Describing macrophage activation states through a relatively extreme binary classification framework based on cellular metabolic differences—where macrophages can polarize into classically (M1) or alternatively (M2) activated subsets—proves highly valuable for understanding immune-metabolic functions in obesity. Although macrophage polarization retains a degree of intrinsic plasticity, these phenotypic states cannot undergo unrestricted or fully reversible interconversion. Instead, they are strongly shaped by local microenvironmental, nutritional, and inflammatory signals, further driving the dynamic progression of obesity-related metabolic disorders across diverse tissues and disease stages.

This review summarizes the classification, functions, and core metabolic regulatory features of macrophages, with a focus on how tissue-resident macrophages sense and respond to metabolic cues in obesity-associated diseases. By integrating current advances in macrophage immunometabolism, we aims to provide theoretical insights for developing novel therapeutic strategies.

## 2 Origins, polarization and phenotypes of macrophages

Macrophages initiate pro-inflammatory responses by recognizing and phagocytosing pathogens, apoptotic cells, and foreign materials, while also mediating anti-inflammatory tissue repair and immunoregulation to sustain tissue homeostasis (6). Monocytes originate from bone marrow hematopoietic stem and progenitor cells via the mononuclear myeloid lineage. Upon release into the circulation, monocytes are recruited to diverse tissues, where they respond to local microenvironmental cues and terminally differentiate into tissue macrophages with distinct polarization profiles (7). Tissue-resident macrophages (such as Kupffer cells, bronchoalveolar macrophages, microglia, and osteoclasts) display significant heterogeneity and maintain homeostasis by performing crucial tissue-specific functions (8). Most tissue-resident macrophages originate from specific yolk sac-derived progenitors in developing embryos and sustain themselves through self-renewal, though they can also be supplemented by differentiation of bone marrow-derived monocytes (9). Monocyte-derived macrophages, however, are primarily recruited to damaged tissues during injury or inflammation (10).

As highly plastic immune cells, macrophages respond to stimuli such as pathogenic microorganisms, cytokines, fatty acids, and their metabolites within distinct local metabolic and immune environments, exhibiting heterogeneous functional and polarization states. Under normal circumstances, naive macrophages (M0) remain non-polarized. Upon exposure to microenvironmental cues, a “metabolic switch” is activated, reprogramming their metabolic profiles and functional phenotypes to adapt to local signals. Based on their activation states and roles in inflammation, macrophages are broadly categorized into classically activated pro-inflammatory M1-like and alternatively activated anti-inflammatory M2-like phenotypes. Stimulation by lipopolysaccharide (LPS) and interferon-gamma (IFN-γ) drives M1 polarization, activating signaling cascades via pattern recognition receptors (PRRs) such as TLRs and NLRs (11). M1 macrophages secrete pro-inflammatory cytokines including tumor necrosis factor-alpha (TNF-α), interleukin-6 (IL-6), interleukin-1β (IL-1β), and inducible nitric oxide synthase (iNOS), exhibiting enhanced phagocytic activity and antigen presentation to clear pathogens, combat tumors, and promote inflammation. M2 macrophages, induced by interleukin-4 (IL-4) and interleukin-13 (IL-13) released from TH2 cells, produce substantial anti-inflammatory cytokines such as IL-10, IL-1 receptor antagonists, and transforming growth factor-β (TGF-β). These macrophages express key genes including arginase 1 (Arg1) and Ym-1, primarily engaging in inflammation suppression, tissue repair, immunoregulation, and defense against helminths and parasitic infections (12). Maintaining the M1/M2 balance is essential for immune homeostasis and normal physiology. In obesity, this balance is disrupted, with an increased proportion of M1 macrophages secreting excessive pro-inflammatory factors. These factors target adipocytes, hepatocytes, and myocytes, interfering with insulin signaling and inducing insulin resistance (13). Moreover, they recruit additional immune cells into adipose tissue and other metabolic organs, amplifying local inflammation and establishing a vicious cycle that drives obesity-associated metabolic disorders. Thus, M1 macrophage overactivation is a key driver of chronic metabolic inflammation.

**Figure 1:**
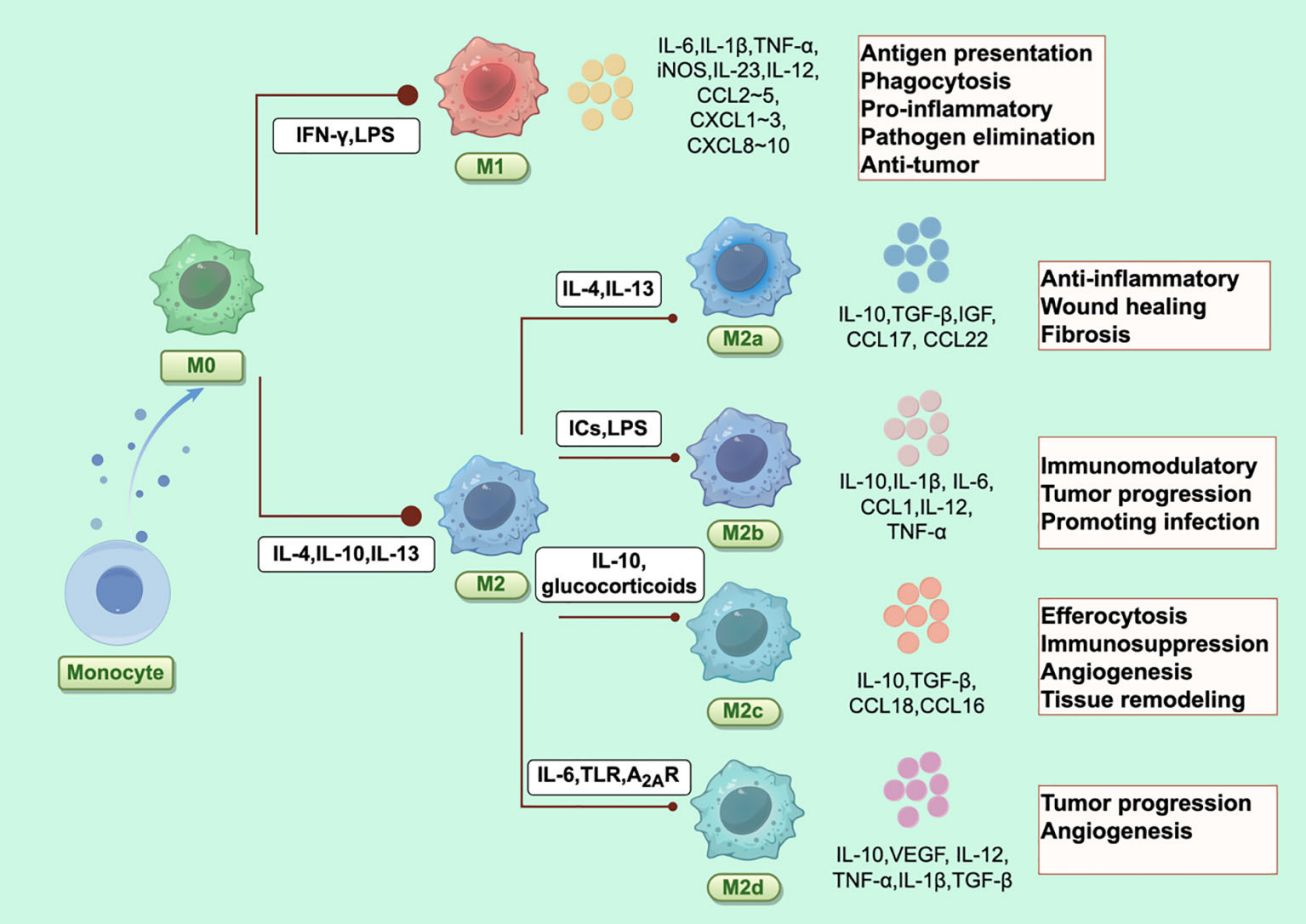
Phenotype and function of macrophage polarization.

Beyond the M1/M2 dichotomy, macrophages exhibit remarkable functional diversity driven by distinct transcriptional programs and stimuli. M2-like macrophages can be further subdivided into M2a, M2b, M2c, and M2d subtypes (**Figure 1****).** These subtypes share the common feature of secreting high levels of the anti-inflammatory cytokine IL-10 and low or undetectable levels of IL-12 (14–16). Most M2 subtypes possess anti-inflammatory and pro-angiogenic properties, secreting growth factors (VEGF, PDGF) and matrix metalloproteinases (MMP2, MMP9) that promote tumor progression (17). M2a (wound-healing macrophages) are induced by IL-4 or IL-13. They suppress excessive inflammation, promote T cell activation and angiogenesis, and secrete profibrotic factors (fibronectin, IGF, TGF-β) to drive tissue remodeling (18). M2b (regulatory macrophages) are activated by LPS or immune complexes via TLR4/Fc receptors. They produce both anti-inflammatory (IL-10, CCL1) and pro-inflammatory cytokines (IL-6, IL-1β, TNF-α, IL-12), functioning to attenuate immune responses while facilitating infection and tumor progression (19). M2c (acquired deactivation macrophages) are induced by glucocorticoids or IL-10-dependent M-CSF signaling. They release high levels of IL-10, CCL18, CCL16, and TGF-β, exhibiting immunosuppressive effects. Characterized by high Mer tyrosine kinase (MerTK) expression, they efficiently phagocytose apoptotic cells and promote proliferation and angiogenesis (17, 20). M2d (tumor-associated macrophages) are polarized by IL-6, TLRs, and adenosine receptors (A2R). They highly express IL-10, TGF-β, and VEGF while showing low expression of IL-12, TNF-α, and IL-1β, thereby promoting tumor angiogenesis, metastasis, and immune tolerance (21, 22). The regulatory balance between M1-like and M2-like phenotypes closely govern immune responses and disease progression.

## 3 Metabolic reprogramming of macrophages

Macrophages serve dual roles as inflammatory mediators and regulators of immune homeostasis. As key sentinels, macrophages rapidly adapt their metabolic pathways to meet functional demands. Phagocytosis, secretion, and migration are energy-demanding, and their metabolic status is dynamically reprogrammed to support phenotypic activation. In obesity, elevated free fatty acids and a chronic inflammatory state drive immunometabolic reprogramming in macrophages. Under inflammatory conditions, enhanced glycolysis promotes M1 polarization, providing energy for inflammatory responses (23). The expression of glucose transporter-1 (GLUT-1) drives the pro-inflammatory phenotype macrophages by increasing glucose uptake, subsequently enhancing glucose metabolism (24). Furthermore, enhanced pentose phosphate pathway generates abundant nicotinamide adenine dinucleotide phosphate (NADPH), providing substrate for reactive oxygen species (ROS) production and thereby exacerbating inflammatory responses (25). In contrast, oxidative phosphorylation and fatty acid β-oxidation support M2 macrophages, maintaining anti-inflammatory and tissue repair functions at low glycolytic levels (23).

In recent years, the heterogeneity and pleiotropic functions of macrophages in health and disease have gained increasing recognition. Macrophages perform distinct roles across different tissues, yet diverse subsets can also participate in the same disease processes. Their recruitment and persistence at pathological sites are closely linked to inflammatory diseases. Macrophages reprogram their metabolic pathways in response to microenvironmental signals—including immune responses, inflammation, tissue damage, nutritional changes, and intercellular interactions—to meet dynamic energy and biosynthetic demands. In obesity-related metabolic disorders, these metabolic alterations not only affect intrinsic macrophage function but also drive disease initiation and progression through crosstalk with other metabolically active cells. Therefore, understanding macrophage metabolic regulation is critical for unraveling the pathogenesis of these disorders.

## 4 Metabolic regulation of different macrophage phenotypes

Macrophage metabolism encompasses nutrient uptake, utilization, and energy production to support cell survival, proliferation, and function. Diverse intra- and extracellular signals remodel metabolic programs that sustain biosynthesis and critically shape immune function (26). Microenvironmental cues drive metabolic reprogramming to orchestrate macrophage activation and polarization, underlying their functional plasticity. Crosstalk among signaling cascades and metabolic pathways (glucose, lipid, and amino acid metabolism) reciprocally influences transcriptional and epigenetic events, leading to distinct functional states (27).In obesity, macrophages encounter a nutrient-saturated microenvironment with elevated fatty acids and glucose, triggering metabolic reprogramming characterized by increased lipid uptake and pathway shifts that profoundly impact macrophage function and inflammation (28). This review summarizes advances in macrophage immunometabolism and key metabolic alterations during phenotypic switching in obesity-associated metabolic diseases.

### 4.1 Glucose metabolism

In resting immune cells—including macrophages—ATP is primarily generated through oxidative phosphorylation (OXPHOS) to sustain basic cellular functions. Glycolysis converts glucose to pyruvate and can proceed under either aerobic or anaerobic conditions. Under normoxia, pyruvate typically enters mitochondria for conversion to acetyl-CoA and subsequent TCA cycle oxidation. However, upon activation, M1 pro-inflammatory macrophages undergo a metabolic switch toward aerobic glycolysis—the “Warburg effect”—preferentially converting glucose to lactate even when oxygen is abundant (29, 30).

**Figure 2:**
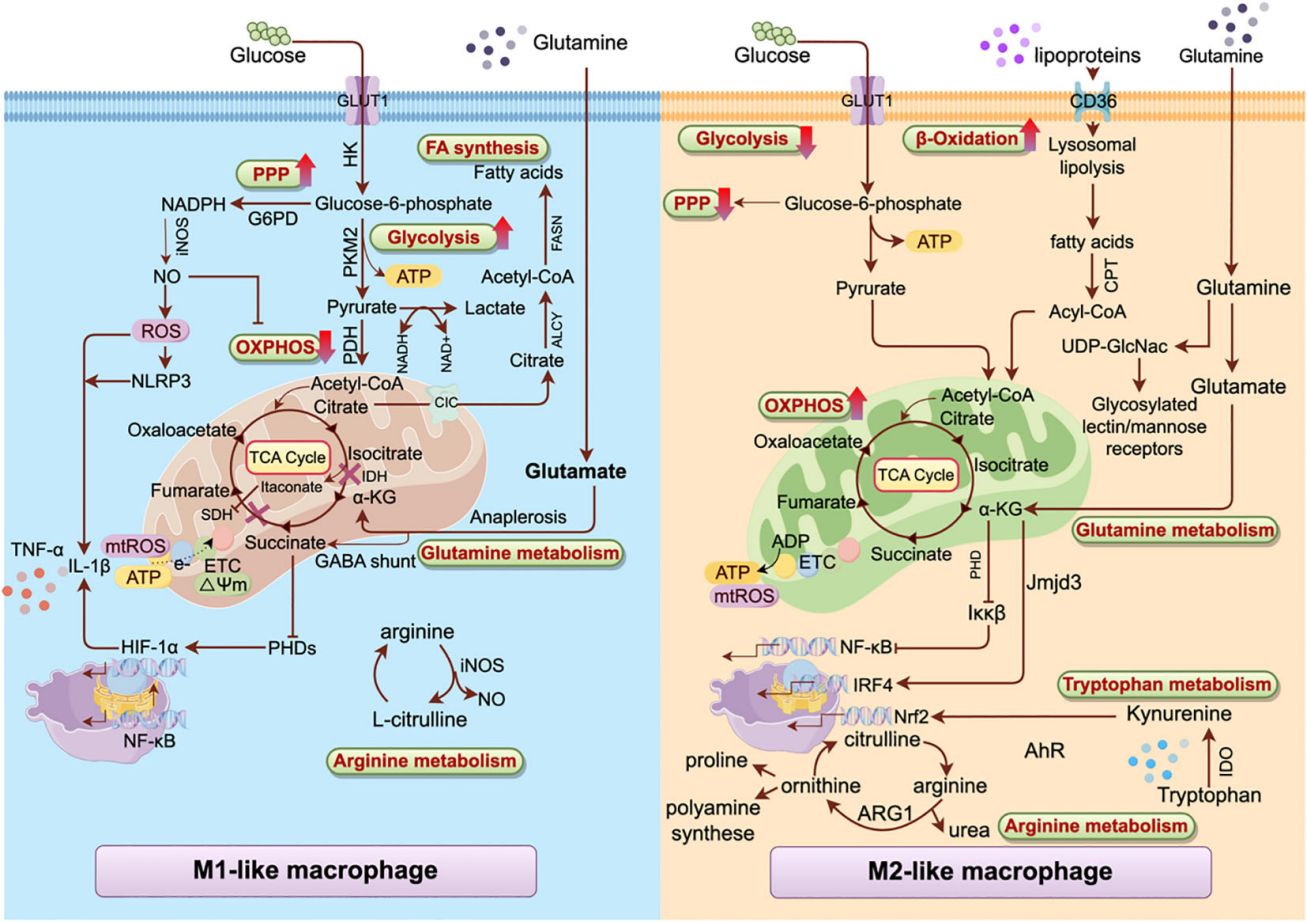
Metabolic Regulation Characteristics of Macrophages. In pro-inflammatory (M1-like) macrophages: Aerobic glycolysis is enhanced, converting glucose to lactate, while PPP flux increases to generate NADPH for ROS and NO production. The TCA cycle is disrupted, leading to accumulation of succinate and citrate and reduced OXPHOS. Upregulation of the GABA shunt or glutaminolysis fuels glutamine anaplerosis, further elevating succinate levels. Succinate in turn stabilizes HIF-1α by inhibiting PHDs, thereby inducing pro-inflammatory and glycolytic gene expression. Additionally, arginine is converted to NO and L-citrulline via iNOS. In anti-inflammatory (M2-like) macrophages: Fatty acid oxidation (FAO) is induced, breaking down fatty acids into pyruvate and acetyl-CoA to fuel the TCA cycle and drive OXPHOS as the primary energy pathway. Glycolysis and PPP activity are markedly reduced. Glutamine is catabolized to α-ketoglutarate (α-KG), replenishing TCA intermediates; α-KG exerts anti-inflammatory effects by modulating PHD activity and inhibiting NF-κB signaling. Arginine is converted to ornithine and polyamines by ARG1, with ornithine serving as a precursor for proline synthesis. Arrows: Red = increased flux/activity; Blue = decreased flux/activity.

M1 macrophages display three key metabolic features: enhanced glycolysis, increased pentose phosphate pathway (PPP) flux, and impaired TCA cycle/OXPHOS (**Figure 2**) (31–33). Aerobic glycolysis produces ATP more rapidly than OXPHOS, albeit with lower efficiency, yielding a net gain of 2 ATP and 2 NADH per glucose molecule. This pathway also generates metabolic intermediates that support nucleotide, amino acid, and fatty acid biosynthesis, as well as NADPH production via the PPP, thereby fueling rapid cellular proliferation and effector functions (34). GLUT1 is a key rate-limiting factor for M1 polarization. GLUT1 overexpression increases glucose uptake and glycolysis, elevates PPP intermediates, reduces oxygen consumption, and drives ROS-mediated pro-inflammatory cytokine production (e.g., IL-1β, IL-6, TNF-α) (24, 35). This ROS burst is essential for the phagocytic activity of M1 macrophages. Consistently, pharmacological inhibition of glycolysis attenuates these pro-inflammatory responses. Of note, while some studies reported that GLUT1 overexpression in certain macrophage cell lines increases glycolytic flux without further inducing IL-6 expression (36), the preponderance of evidence supports a causal role for enhanced glycolysis in driving M1 pro-inflammatory polarization.

Hypoxia-inducible factor-1α (HIF-1α) is a master regulator of this glycolytic switch (37). Under inflammatory conditions, NF-κB activation not only drives pro-inflammatory gene expression but also induces HIF-1α transcription (38, 39). The stability of HIF-1α is further enhanced by TCA cycle intermediates—including succinate, fumarate, and citrate—which inhibit prolyl hydroxylase (PHD) activity (40). Once stabilized, HIF-1α upregulates the expression of GLUT1, glycolytic enzymes, and pyruvate dehydrogenase kinase 1 (PDK1) (41). PDK1 in turn phosphorylates and inhibits pyruvate dehydrogenase (PDH), diverting pyruvate from the TCA cycle toward lactate production, thereby sustaining glycolytic flux (42, 43). Notably, inhibiting the HIF-1α-PDK1 axis has been shown to suppress systemic inflammation and reduce macrophage migration (44).

The role of glycolysis in M2 macrophage activation remains debated. While M2 macrophages are classically associated with OXPHOS, some studies report that IL-4 stimulation can also upregulate glycolysis, mediated by mTORC2-IRF4 or mTORC1 signaling (45, 46). However, this glycolytic increase may be permissive rather than instructive. Using the glycolytic inhibitor 2-deoxy-D-glucose (2-DG), Tan et al. found that PDK1 knockdown suppresses M1 activation but paradoxically enhances IL-4-induced the expression of M2 macrophage markers such as Arg1, YM-1, FIZZ-1, and MRC1 (47). Conversely, Wang et al. demonstrated that 2-DG impairs both glycolysis and OXPHOS, reducing ATP levels and JAK-STAT6 signaling and thereby compromising M2 differentiation. Critically, M2 differentiation proceeds normally when OXPHOS remains intact, even in the absence of glycolytic stimulation (48). Under steady-state conditions, OXPHOS—not glycolysis—is the dominant metabolic pathway across tissue macrophage (49). Thus, targeting OXPHOS may offer a more selective therapeutic strategy for macrophage-related metabolic diseases.

In the context of obesity, sustained exposure to elevated glucose and free fatty acids reinforces the glycolytic bias of adipose tissue macrophages (ATMs), promoting their M1-like polarization and contributing to systemic insulin resistance. These metabolic insights position glycolysis and its regulatory nodes—HIF-1α, PDK1, and GLUT1—as promising therapeutic targets for obesity-associated metabolic disorders.

### 4.2 Tricarboxylic acid cycle

The TCA cycle is driven by acetyl-CoA reactions, through which NAD^+^ and FAD are reduced to NADH and FADH_2_ in a series of enzymatic reactions. These coenzymes subsequently transfer electrons to the electron transport chain (ETC), enabling OXPHOS and efficient ATP production with oxygen participation. The TCA cycle serves as the central hub regulating macrophage energy metabolism and function. In M1 macrophages, although aerobic glycolysis is the primary energy source, the TCA cycle undergoes multiple interruptions that restrict overall flux. These interruptions lead to the accumulation and mitochondrial escape of specific intermediate metabolites—including citrate, itaconate, and succinate—which subsequently acquire regulatory functions beyond their classical metabolic roles (50).

Two critical breakpoints in the TCA cycle have been identified in pro-inflammatory macrophages. The first breakpoint occurs at isocitrate dehydrogenase (IDH), where inhibited activity causes citrate accumulation and obstructs its conversion to α-ketoglutarate (α-KG), thereby enhancing intracellular lipid synthesis and itaconate production. In the cytosol, citrate is transported via the mitochondrial citrate carrier (CIC) and cleaved by ATP-citrate lyase (ACLY) into acetyl-CoA and oxaloacetate, fueling fatty acid synthesis and providing precursors for pro-inflammatory mediators such as prostaglandins (51, 52). Cytosolic citrate also generates NADPH via ACLY, which is utilized by NADPH oxidase (NOX) and iNOS to produce ROS and NO, respectively, thereby amplifying inflammation (52). In mitochondria, however, citrate-derived itaconate exerts anti-inflammatory effects by activating Nrf2 (which suppresses IL-1β transcription), inducing glutathione and ATF3 (which reduce ROS levels), and negatively regulating pro-inflammatory cytokines (53). The second breakpoint is mediated by itaconate, which accumulates and inhibits succinate dehydrogenase (SDH) activity, leading to elevated succinate levels (54–56). Beyond its intracellular role, succinate released into the extracellular milieu binds to its cognate receptor SUCNR1 (GPR91), further upregulating IL-1β and creating an autocrine amplification loop that sustains inflammation (57, 58). α-KG acts as a counterbalancing metabolite. Derived from glutamine metabolism, α-KG competitively inhibits PHD activity at high concentrations, stabilizing HIF-1α while preventing TCA cycle stagnation. Research indicates that a low α-KG/succinate ratio enhances M1 macrophage activation, while a high α-KG/succinate ratio promotes the M2 phenotype (59). Furthermore, M2 macrophages often rely on glutamine metabolism to supply energy and biosynthetic precursors. Inhibiting glutamine synthetase shifts M2 macrophages toward an M1 phenotype. This shift is characterized by intracellular glutamine depletion and compensatory enhancement of glycolysis leading to succinate accumulation, which promotes HIF-1α activation (60).

Mitochondrial dysfunction further amplifies this pro-inflammatory program. LPS-stimulated macrophages exhibit elevated mitochondrial membrane potential (ΔΨm), which synergizes with SDH-driven mitochondrial oxidation to promote reverse electron transport (RET)—a process in which electrons flow backward through complex I. RET drives mitochondrial ROS (mtROS) production at complex I, ultimately inducing IL-1β generation (61, 62). mtROS in turn activates MAPK and NF-κB signaling to promote pro-inflammatory gene expression (63) and stabilizes HIF-1α, reinforcing the glycolytic bias and IL-1β production in inflammatory macrophages (64).

Collectively, the TCA cycle in M1 macrophages exhibits a “low-throughput, pro-inflammatory intermediate accumulation” profile. This metabolic framework sustains basal cycling through glutamate/glutamine metabolism while simultaneously amplifying inflammatory signaling via metabolites such as succinate and α-KG. This paradigm prioritizes rapid glycolytic energy supply while repurposing TCA cycle intermediates for immune regulation, represents an adaptive strategy that supports pro-inflammatory macrophage functions. Understanding this metabolic logic opens therapeutic opportunities for chronic inflammatory diseases (e.g., obesity-related metabolic disorders), such as targeting the succinate-HIF-1α axis or modulating glutamate metabolism to curb excessive inflammation.

Unlike M1 metabolism, M2 macrophages exhibit high and intact TCA cycle activity. Following glucose uptake, a portion is utilized for glycogen and fatty acid synthesis to store energy, while pyruvate preferentially enters mitochondria to participate in the TCA cycle. This process generates substantial ATP through OXPHOS, providing ample energy for cellular anabolic metabolism and tissue repair functions. M2 macrophages exhibit high dependence on glutamine metabolism, providing partial substrates for the TCA cycle (50). Following glutaminolysis to glutamate, further conversion to α-KG significantly replenishes TCA cycle intermediate metabolites, sustaining cycle flux to meet high energy demands during anti-inflammatory states.

This metabolic advantage of M2 macrophages is, however, compromised under obese conditions. Beyond promoting TCA cycle intermediate accumulation, obesity induces profound alterations in mitochondrial activity and ETC function in macrophages. Lean adipose tissue macrophages maintain anti-inflammatory phenotypes via OXPHOS through intact ETC complexes. However, in the obese state, adipose tissue macrophages exhibit reduced OXPHOS and impaired ETC function, accompanied by increased glycolytic flux (65, 66). Mechanistically, obesity stabilizes HIF-1α, which upregulates glycolytic enzymes (GLUT1, PGK1, PKM2) and suppresses ETC Complexes I and III (67). This ETC dysfunction promotes electron leakage and mtROS overproduction, which in turn exacerbate pro-inflammatory signaling via NF-κB and MAPK activation (68). Thus, a self-reinforcing loop emerges in which impaired mitochondrial respiration drives glycolytic reprogramming and M1 polarization, while pro-inflammatory signals further exacerbate ETC dysfunction.

### 4.3 Pentose phosphate pathway

The pentose phosphate pathway (PPP) is upregulated in M1 macrophages and contributes to their pro-inflammatory responses (50). Glucose is initially converted to glucose-6-phosphate (G6P) through the hexokinase reaction, utilizing ATP. G6P can be metabolized via the glycolytic pathway to generate ATP for sustaining cellular function, and can also undergo esterification to produce lipids (triglycerides and phospholipids) (69). Glycolysis also facilitates the channeling of carbon flux into the oxidative PPP. During the oxidative phase, NADP^+^ is reduced to NADPH, which serves as a coenzyme for iNOS catalytic reactions to facilitate NO production and donates electrons to NADPH oxidase for ROS generation to eliminate pathogens (70, 71).In the non-oxidative phase, glycolytic intermediates are diverted to synthesize ribose-5-phosphate and amino acids. ROS play a crucial role in activating and sustaining M1 macrophages. Studies reveal that PPP restriction significantly reduces intracellular ROS production while sensitizing macrophage polarization toward the M2 (72). In obesity and diabetes, hyperglycemia-induced ROS promotes M1-like polarization (72). Glucose-6-phosphate dehydrogenase (G6PD) is the rate-limiting enzyme of the PPP and a key regulator of cellular redox balance. G6PD promotes ROS production under stress conditions (73), and its suppression in macrophages attenuates pro-inflammatory responses (74).

Research has revealed G6PD expression is elevated in adipose tissue macrophages of obese animals and positively correlates with pro-inflammatory gene expression. This upregulation promotes oxidative stress and inflammation, potentially through enhanced activation of the p38 MAPK and NF-κB pathways (75). Thus, targeting G6PD activity represents a potential strategy to modulate macrophage function in metabolic diseases. The PPP is also controlled by carbohydrate kinase-like protein (CARKL; sedoheptulose kinase), which catalyzes sedoheptulose-7-phosphate production and modulates carbon flux in the non-oxidative phase, thereby influencing NADPH generation (76). In LPS-activated macrophages, CARKL expression is reduced, leading to increased PPP flux and pro-inflammatory cytokine production. Conversely, IL-4-activated macrophages upregulate CARKL, suppressing PPP activity, reducing NADPH and ROS, and promoting an anti-inflammatory phenotype (76). Therefore, downregulation of CARKL appears crucial for redirecting glucose flux from aerobic metabolism toward glycolysis and the PPP in pro-inflammatory macrophages.

### 4.4 Lipid metabolism

#### 4.4.1 Fatty-acid synthesis

Beyond alterations in glucose metabolism, fatty acid metabolism also undergoes reprogramming following immune cell activation. The FAS pathway represents a crucial cellular process essential for membrane biosynthesis and energy storage, with fatty acid synthesis being closely associated with the pro-inflammatory effects and signal transduction functions of macrophages (77). In M1 macrophages, fatty acid synthesis (FAS) is markedly enhanced. Mechanistically, citrate accumulation from TCA cycle disruption activates acetyl-CoA carboxylase (ACC), which converts acetyl-CoA to malonyl-CoA—a key precursor for FAS. Malonyl-CoA also inhibits carnitine palmitoyltransferase I (CPT1), thereby blocking fatty acid entry into mitochondria and suppressing fatty acid oxidation (FAO) (78). In obesity, elevated circulating free fatty acids are taken up by macrophages via CD36 and fatty acid transport proteins (FATPs) (79, 80). After internalizing substantial fatty acids, macrophages partially utilize them to synthesize lipids including triglycerides and cholesterol, storing these within lipid droplets. As lipid accumulation progresses, macrophages transform into foam cells, a hallmark of obesity-related metabolic diseases such as atherosclerosis.

During M1 macrophage differentiation of human monocytes stimulated by macrophage colony-stimulating factor (M-CSF), key transcription factors SREBPs (sterol regulatory element-binding proteins) for fatty acid synthesis are upregulated. This process is essential for pseudopodia formation and organelle development in macrophages, demonstrating that FAS is critical for macrophage development and phagocytic activity (81). Researchers have discovered that SREBP-1a not only activates lipogenic genes but also induces Nlrp1a expression, a key component of inflammasome that promotes IL-1β release (82). The pro-inflammatory response in macrophages can be suppressed by inhibiting fatty acid synthase (FASN), a key enzyme of FAS that catalyzes the production of long-chain fatty acids (83). Consistently, myeloid-specific FASN deficiency prevents adipose tissue macrophage recruitment and inflammation in mice (84). Liver X receptor (LXR) signaling regulates macrophage cholesterol metabolism and also influences anti-pathogen responses (85). Activation of LXR promotes M2 macrophage polarization (86), and LXR-α expression suppresses M1 responses and inflammation by inhibiting NF-κB and AP-1 activity while also reducing apoptosis (87).

#### 4.4.2 Beta oxidation

In contrast to the glycolytic metabolism of M1 macrophages, IL-4-induced alternative activation is characterized by enhanced lipid uptake and oxidation (88, 89). Internalized lipids are catabolized in lysosomes to free fatty acids, which are then transported into mitochondria for β-oxidation. FAO supplies acetyl-CoA to the TCA cycle and fuels OXPHOS, providing a sustained energy source for M2 functions (90). Studies have revealed that IL-4 stimulation upregulates STAT6, PPARγ, and PGC-1β, driving FAO and mitochondrial biogenesis. M2 macrophages express higher levels of CPT1, CD36, and medium-chain acyl-CoA dehydrogenase (MCAD) while suppressing pro-inflammatory cytokines (23). PPARγ is essential for the maturation of alternatively activated macrophages. Mice with macrophage-specific PPARγ deficiency exhibit impaired M2 activation, rendering them more susceptible to diet-induced obesity, insulin resistance, and glucose intolerance (91). PPARγ also promotes alternative macrophage activation by promoting glutamate metabolism (92). Glutaminolysis-derived α-KG enhances M2 polarization through Jmjd3-dependent epigenetic regulation and suppresses M1 inflammation by inhibiting the NF-κB pathway (59).

However, considerable debate exists regarding whether FAO is essential for macrophage polarization into the M2 phenotype. Although CPT1 is the rate-limiting enzyme for FAO, several lines of evidence question its necessity. First, high-dose etomoxir (a CPT1 inhibitor) blocks IL-4-induced M2 polarization, but this effect may be due to depletion of intracellular free CoA rather than specific FAO inhibition (93). Second, macrophages from CPT2-deficient mice—which have impaired FAO—retain the capacity to polarize into the M2 phenotype upon IL-4 stimulation (94). Third, FAO inhibition does not affect IL-4-induced gene expression in human macrophages, suggesting species-specific metabolic requirements (95). Collectively, these findings indicate that FAO is dispensable for M2 activation. Instead, FAS may support M2 polarization by providing fuel for OXPHOS (45). Thus, FAO and its metabolites play complex, context-dependent roles in macrophage reparative functions and inflammation.

Beyond mitochondria, peroxisomes also carry out very-long-chain fatty acid (VLCFA) β-oxidation and lipid mediator synthesis, which modulate macrophage inflammatory responses. Peroxisomes form multi-organelle units (mitochondria-ER-peroxisome-lipid droplet clusters) that coordinate inflammatory lipid metabolism. In this context, PPAR signaling in adipose tissue macrophages emerges as an additional node linking peroxisomal function to systemic metabolic improvement in obesity (96, 97). Nevertheless, compared with the well-established mitochondrial ETC-HIF-1α-glycolysis axis, the relative contribution of peroxisomal dysfunction to M1 macrophage polarization remains less defined.

### 4.5 Amino acid metabolism

#### 4.5.1 Glutamine metabolism

Glutamine metabolism is essential for energy supply and functional maintenance in macrophages, exhibiting distinct metabolic fates in M1 and M2 macrophages. Glutamine can be converted to glutamate via glutaminase, then synergistically produces succinate through either the γ-aminobutyric acid (GABA) shunt pathway or participation in the TCA cycle, thereby providing cellular energy.

In M1 macrophages, glutamine is converted to glutamate via glutaminase, and subsequently to succinate through the γ-aminobutyric acid (GABA) shunt or TCA cycle anaplerosis. This succinate accumulation stabilizes HIF-1α and promotes IL-1β production, as detailed in Section 4.2 (54). Glutamine also contributes to glutathione synthesis and serves as a nitrogen source for nucleotides and amino acids, supporting macrophage activation and inflammatory responses (98).

In M2 macrophages, glutamine metabolism is critical for polarization. Transient glutamine deprivation does not affect M1 markers but markedly suppresses M2 marker expression (Arg1, Mrc1, Ym1, Retnla) and reduces TCA cycle activity (50, 99). Mechanistically, glutamine provides the essential substrate for uridine diphosphate N-acetylglucosamine (UDP-GlcNAc) synthesis—a key metabolite for protein glycosylation and signaling. Tracer studies have shown that approximately one-third of carbon in TCA cycle metabolites and over half of nitrogen in UDP-GlcNAc originate from glutamine in M2 macrophages (50). Moreover, M2 macrophage glutamine metabolism does not rely entirely on exogenous uptake but can autonomously synthesize glutamine from glutamate and ammonia via glutamine synthetase (GS).

Collectively, these findings establish that glutamine metabolism is indispensable for M2 polarization, whereas M1 macrophages rely less on glutamine. Understanding this differential dependency may inform strategies to selectively modulate macrophage phenotypes in obesity-associated metabolic diseases.

#### 4.5.2 Arginine metabolism

L-arginine, a key amino acid regulating macrophage activation, can be synthesized via citrulline intermediates dependent on glutamine metabolism. Specifically, the metabolic flux catalyzed by iNOS, which converts L-arginine into NO, promotes macrophage polarization toward the M1 phenotype. Conversely, the metabolic pathway catalyzed by Arginase (Arg1) regulates macrophage polarization toward the M2 phenotype (100).

Under pro-inflammatory stimulation with LPS, TNF-α or IFN-γ, M1 macrophages highly express iNOS, an enzyme that catalyzes the conversion of arginine to NO and L-citrulline. NO exerts antibacterial and antiviral effects but also amplifies pro-inflammatory cytokine production (IL-1β, TNF-α). Excessive NO exacerbates tissue damage and inflammation. Notably, iNOS blockade allows LPS/IFN-γ-treated M1 macrophages to repolarize toward an M2 phenotype upon IL-4 stimulation, indicating that NO impedes M1-to-M2 repolarization (33).

M2 macrophages exhibit high expression of Arg1, which catalyzes the hydrolysis of arginine into urea and L-ornithine. The product L-ornithine is further metabolized by ornithine decarboxylase (ODC) into polyamines and proline, which regulate cell proliferation and collagen synthesis, thereby facilitating tissue repair and immune suppression (51). PPAR transcription factors regulate the expression of Arg1 in M2 macrophages. This pathway likely represents the primary mechanism through which tissue-resident macrophages (such as adipose tissue macrophages) acquire the M2-like Arg1^+^ phenotype. Through M2-like metabolic pathways, these macrophages exert an anti-inflammatory balance in adipose tissue and systemic metabolism (101, 102). Furthermore, Arg1 competes with iNOS for the substrate arginine. Numerous pathogens exploit this mechanism by upregulating arginase expression to restrict arginine availability for iNOS, thereby suppressing NO production (103).

#### 4.5.3 Tryptophan metabolism

Tryptophan metabolism influences macrophage function and polarization through the kynurenine pathway. Tryptophan is catabolized into kynurenine (Kyn) by indoleamine 2,3-dioxygenase (IDO) or tryptophan 2,3-dioxygenase (TDO), and can be further metabolized to downstream products such as quinolinic acid (104, 105). IDO expression is induced by IFN-γ, TNF-α, or prostaglandins. Overexpression of IDO promotes M2 polarization, whereas IDO silencing shifts macrophages toward a pro-inflammatory phenotype (106). Mechanistically, Kyn activates the aryl hydrocarbon receptor (AhR), which inhibits NF-κB signaling, reduces pro-inflammatory cytokine secretion, and favors an anti-inflammatory state (107). In tumor-associated macrophages (TAMs), high IDO expression leads to AhR activation, suppressing Th17 cells and inducing regulatory T cells (Tregs) to promote immune escape (108). Another tryptophan-catabolizing enzyme, interleukin-4-induced gene 1 (IL4I1), is upregulated during BMDM differentiation. IL4I1 degrades L-tryptophan and enhances the M2 phenotype, likely through STAT-6 and STAT-3 phosphorylation (109). Collectively, tryptophan metabolism—primarily via the IDO–Kyn–AhR axis—supports M2 polarization and resolution of inflammation, though the relative contribution of this pathway in obesity-associated metabolic diseases remains to be fully elucidated.

## 5 Role of macrophages and crosstalk with target cells in obesity-related metabolic diseases

Chronic low−grade inflammation drives obesity, insulin resistance, and T2DM (110). Macrophages serve as key effectors in this process, triggering inflammation and insulin resistance (111). Obesity-induced insulin resistance in metabolic organs arises from both dysfunction of insulin−responsive cells (adipocytes, hepatocytes, and myocytes) and infiltration of pro-inflammatory macrophages. Macrophage accumulation drives tissue inflammation and fibrosis, thereby exacerbating metabolic disorders. Beyond metabolic alterations influencing functional phenotypes, factors such as cellular origin and tissue localization are now considered significant variables determining macrophage biological functions. Accordingly, the regulation of metabolic programs in macrophage populations across distinct microenvironments, as well as their crosstalk with neighboring cells, remains an active area of investigation.

Beyond the classical M1/M2 polarization framework offers instructive value, it cannot fully encompass the true heterogeneity of macrophages in obesity-associated metabolic diseases (112). Single-cell transcriptomics and spatial multi-omics studies based on human samples have revealed that macrophages in adipose tissue and liver are dynamically shaped by local lipid load, glycolytic flux, and hypoxic signals, giving rise to diverse metabolically defined functional subsets (113). They do not simply align with canonical M1 or M2 signatures but instead undergo stage-dependent, tissue-specific functional switching. Lipid-associated macrophages (LAMs), characterized by high expression of TREM2, CD9, and GPNMB, are prominent in both obese adipose tissue and MASLD-affected liver, where they mediate lipid clearance and tissue remodeling (114, 115). Metabolically activated macrophages (MMe) exhibit elevated glycolysis and fatty acid synthesis without the full classical M1 signature; they are enriched in adipose tissue of obese individuals and contribute to insulin resistance (116, 117). Additionally, certain tissue-resident subsets—such as the protective SerpinB2^+^ population in visceral adipose tissue—play indispensable roles in preserving insulin sensitivity (118). The presence of these subsets underscores the need to move beyond binary classification and consider macrophage phenotypic diversity in the pathogenesis of obesity-associated metabolic diseases.

In the following sections, we discuss how tissue−specific macrophages in adipose tissue, liver, and pancreatic islets interact with neighboring cells to drive metabolic inflammation and disease progression, while highlighting the metabolic features that underlie their functional plasticity.

### 5.1 Macrophages and adipose tissue

**Figure 3:**
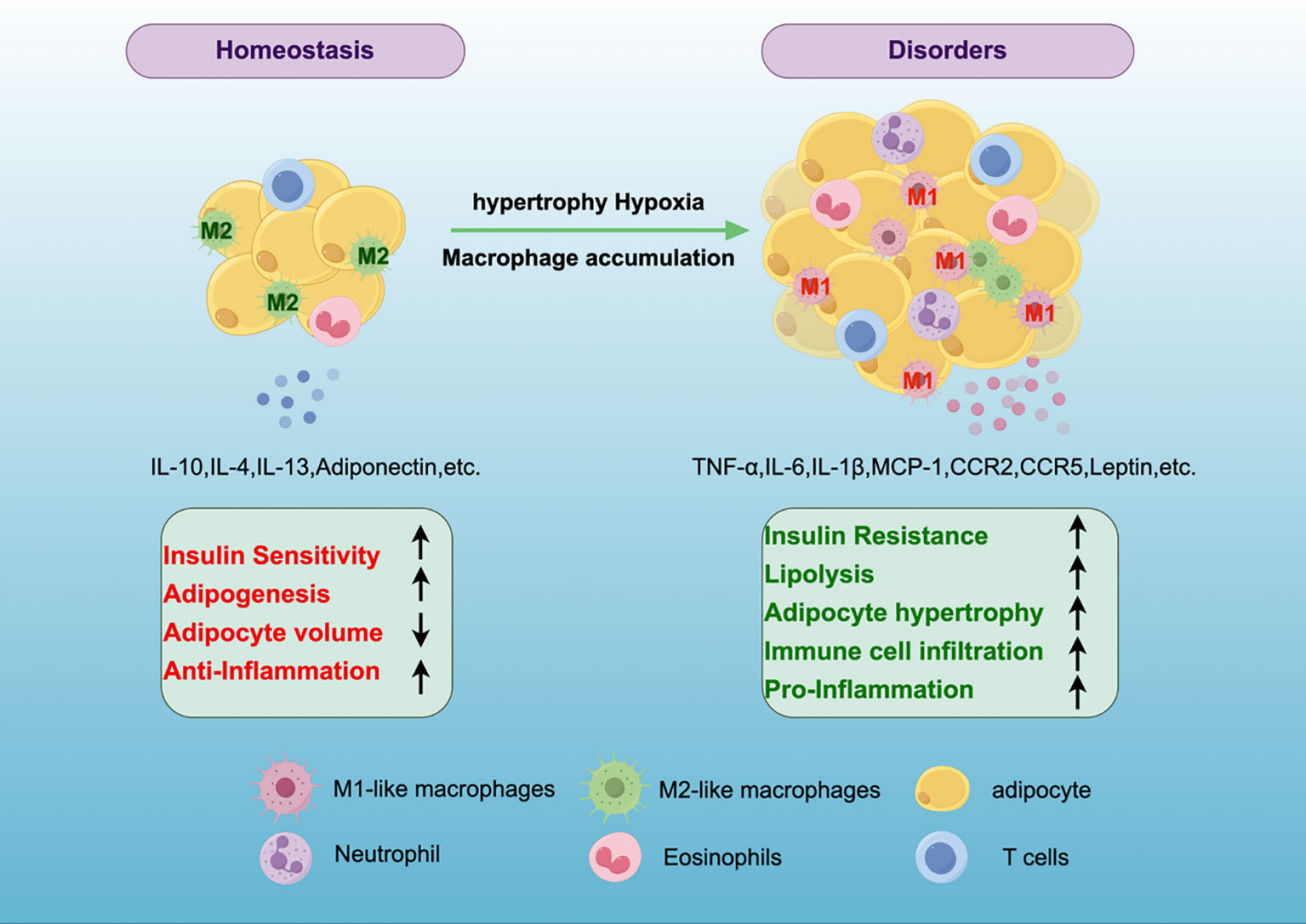
Roles of M1-like and M2-like Macrophages in Adipose Tissue. Under lean conditions, adipocytes secrete factors such as adiponectin, which stimulate the predominance of M2-like macrophages in adipose tissue. These M2-like macrophages produce anti-inflammatory cytokines that enhance insulin sensitivity and reduce systemic inflammation. Additionally, they support adipogenesis during adipose tissue remodeling. In obese adipose tissue, excessive lipid accumulation leads to adipocyte hypertrophy and a hypoxic state. Lipolysis releases free fatty acids and various pro-inflammatory adipokines, which promote macrophage polarization toward the M1-like phenotype. These M1-like macrophages produce pro-inflammatory cytokines that interact with receptors on adipocytes, inhibiting insulin signaling pathways and inducing intracellular insulin resistance. This process further exacerbates metabolic disorders.

In obesity, adipose tissue macrophages (ATMs) are central to chronic inflammation and metabolic dysfunction. Adipose tissue comprises white (WAT), brown (BAT), and beige depots, with WAT being the most abundant and primarily responsible for energy storage. ATMs are the most abundant innate immune cells in obese adipose tissue, increasing from 5-10% of total cells in lean states to over 50% in extreme obesity (119). This indicates that obesity significantly alters the macrophage-to-adipocyte ratio. Functionally, ATMs in obesity predominantly adopt an M1 phenotype, secreting pro-inflammatory cytokines (TNF-α, IL-1β, IL-6), whereas lean adipose tissue is dominated by M2 ATMs producing anti-inflammatory IL-10 and TGF-β, thereby promoting inflammation resolution and tissue homeostasis (**Figure 3**) (120).

With the progression of obesity, ATM polarization shifts from the anti-inflammatory M2 state to the pro-inflammatory M1 phenotype, perpetuating chronic low-grade inflammation characteristic of obesity (121). Nutrient overload triggers adipocyte necrosis and apoptosis. Macrophages cluster around dead adipocytes to clear debris, forming crown-like structures (CLS) (119, 122). Macrophages within CLS store and buffer excess lipids, becoming lipid-laden foam macrophages (123). The number of CLS correlates positively with adipose tissue inflammation and metabolic dysfunction (124). Using single-cell transcriptomics and spatial transcriptomics of murine adipose tissue, Jaitin et al. identified LAMs as a distinct subset characterized by high expression of *Trem2*, *Cd9*, *Lpl*, and *Gpnmb* (114). LAMs are also present in human adipose tissue and differ from both M1 and M2 macrophages. Their transcriptional profile includes both pro-inflammatory genes and lipid metabolism-associated genes, reflecting their specialized function in lipid buffering and tissue remodeling. Trem2^+^ deficiency in HFD-fed mice led to phenotypic reversal in AT Trem2^+^ macrophages and reduced lipid accumulation, which was associated with exacerbated adipocyte hypertrophy, systemic lipid accumulation, and glucose intolerance. This highlights that lipid metabolism and accumulation in Trem2^+^ macrophages may play a protective role in obesity-induced metabolic diseases.

HIF-1α is activated in obese adipose tissue, shifting fuel consumption from OXPHOS to glycolysis and promoting M1 polarization (120, 125). M1−derived inflammatory factors act on adipocytes to suppress insulin signaling and induce insulin resistance. In adipose tissue of obese individuals, IL-1β from M1 ATMs activates IKK/NF-κB, leading to serine phosphorylation of IRS-1 and blunting insulin signaling (111). Similar to IKKβ, JNK inhibits insulin signaling via IRS-1 phosphorylation, thereby reducing PI3K/AKT signaling transduction (126, 127). Saturated fatty acids (SFAs) and pro−inflammatory adipokines (e.g., MCP−1, TNF−α) released from hypertrophic adipocytes trigger TLR2/TLR4 signaling in ATMs, activating NF−κB, JNK, and NLRP3 inflammasome assembly, which drives chemokine and cytokine expression and sustains adipose tissue inflammation (128–131). These mechanisms also promote the recruitment and M1 polarization of additional ATMs (132). Inflammation-induced dysfunction of adipocytes accelerates lipid spillover to skeletal muscle and liver, causing ectopic lipid deposition and systemic insulin resistance (110, 133, 134). Additionally, adipocyte-derived microvesicles carrying miR-155 promote M1 ATM polarization, further exacerbating glucose intolerance (135).

Lean adipocytes typically secrete adiponectin, stimulating M2 macrophage polarization (136). Adiponectin is the most abundant adipokine produced by adipocytes; it enhances insulin sensitivity and reduces systemic inflammation, whereas its levels decrease in obese individuals (137). Adiponectin, a well-known AMPK activator, shifts macrophage polarization from M1 to M2 phenotype, thereby suppressing adipose tissue inflammation (138). PPARs are ligand-dependent transcription factors that act as fatty acid sensors in the body, regulating glucose and lipid metabolism (139). Macrophages express multiple free fatty acid receptors, including TLRs, CD36, and G protein-coupled receptors (GPCRs) (140–142). Following receptor binding, SFAs modulate chemokine expression through mechanisms involving ROS generation, NF-κB and PPAR-γ (143). PPAR-δ plays a crucial role in M2 macrophage activation and alleviates diet-induced insulin resistance (144). IL-4 also induces PPAR-δ expression in macrophages, which synergizes with STAT6 to coordinate selectively activated macrophage functions and suppress pro-inflammatory gene expression (145). PPAR-γ has been reported to polarize human monocytes into M2 macrophages *in vitro* (146), whereas myeloid-specific PPAR-γ loss impairs M2 activation and accelerates obesity and insulin resistance in mice (91).

During obesity development, Adipose tissue expansion requires extracellular matrix remodeling and angiogenesis, processes supported by M2-like ATMs. However, rapid adipocyte enlargement outpaces angiogenesis, increasing oxygen consumption and causing local hypoxia, which activates HIF-1α (147). Hypoxia, in turn, induces adipocytes to produce chemokines (MCP-1, LTB4) that recruit immune cells and promote M1 polarization (148). Upregulated CXCL10 and CXCL11 in obese adipose tissue further suppress angiogenesis, exacerbating hypoxia (149). Additionally, macrophages secrete TNF-α, TGF-β, and macrophage-inducible C-type lectin, which suppress adipogenesis and promote adipose tissue fibrosis, ultimately impairing metabolic function (150, 151). Thus, within adipose tissue, macrophages orchestrate lipid and energy metabolism and modulate insulin sensitivity through a network of adipokines and chemokines (152).

### 5.2 Macrophages and liver tissue

Similar to adipose tissue remodeling in obesity, hepatic macrophages undergo polarization, recruitment, and proliferation during NAFLD pathogenesis (153). Hepatic macrophages—comprising embryo-derived Kupffer cells (KCs) and monocyte-derived infiltrating macrophages—are central to the progression from simple steatosis to metabolic dysfunction-associated steatohepatitis (MASH) and fibrosis (154). KCs reside in liver sinusoids and maintain homeostasis by clearing pathogens, phagocytosing cellular debris, regulating iron and lipid metabolism, and preserving immune tolerance (9, 155). Upon metabolic stress, KCs become activated and recruit circulating monocytes, which differentiate into pro-inflammatory macrophages, amplifying liver inflammation (156).

**Figure 4:**
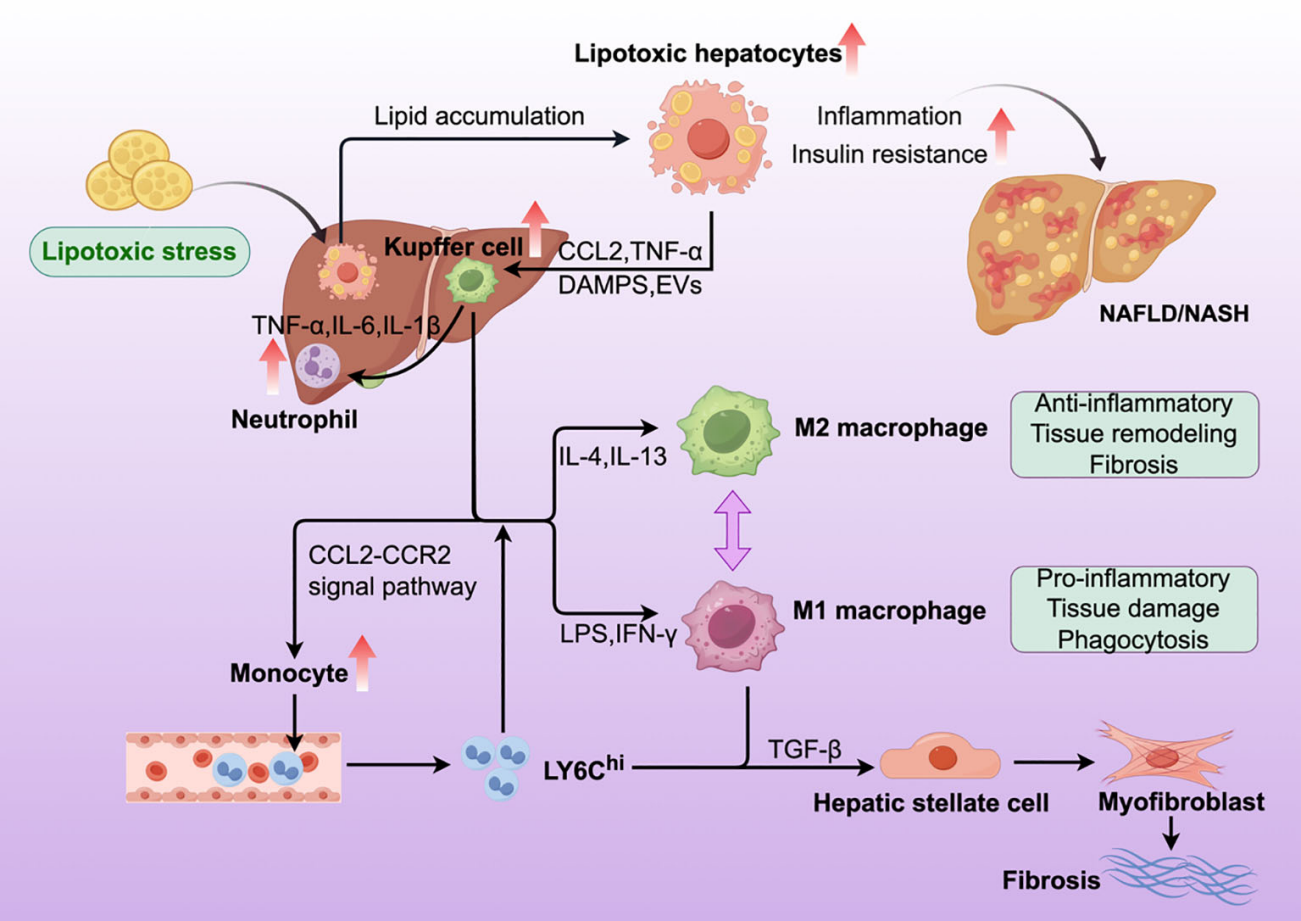
Role of Macrophages in Hepatic Tissue in the Context of Obesity. Lipotoxicity-induced hepatocyte injury triggers the release of cytokines and other signals that activate resident macrophages and recruit monocytes and neutrophils. Early infiltrating monocytes differentiate into pro-inflammatory macrophages, aggravating inflammation and insulin resistance. Cytokines from Kupffer cells and monocyte-derived macrophages subsequently activate hepatic stellate cells (HSCs), promoting their transition to myofibroblasts and driving fibrosis. This process ultimately accelerates the progression from NAFLD to NASH.

Lipid accumulation in hepatocytes induces lipotoxicity, leading to cell dysfunction and death, which can progress to non-alcoholic steatohepatitis (NASH), advanced fibrosis, cirrhosis, and hepatocellular carcinoma (157). Saturated fatty acids and hepatocyte-derived damage signals (CCL2, TNF-α) activate KCs, promoting their M1-like polarization and the recruitment of CCR2^+^Ly6C^+^ monocytes, which differentiate into pro-inflammatory macrophages (**Figure 4**) (158–160). In murine fatty liver models, M1-like macrophages promote triglyceride synthesis via enhanced diacylglycerol acyltransferase activity (161), drive hepatic inflammation through VCAM-1, ICAM-1, and TNF-α (162), and suppress FAO by inhibiting PPAR-α (163). Notably, hepatic macrophage depletion or inhibition of monocyte recruitment prevents steatosis and inflammation in murine NASH models (164). M2-like Kupffer cells ameliorate hepatocyte steatosis and apoptosis in NASH models (165). PPAR-γ/δ can shift KCs toward an M2 phenotype, improving insulin resistance and NAFLD (detailed in Section 4.4) (144, 166). Maternal obesity-induced KCs metabolic reprogramming (HIF-1α-driven glycolysis) predisposes offspring to fatty liver disease (167).

In the steatotic liver, lipid overload drives resident Kupffer cells toward a glycolysis-dominant metabolism, characterized by HIF-1α stabilization and lactate production (168). This metabolic shift is not merely an epiphenomenon but directly instructs pro−inflammatory activation. As detailed in Sections 4.1 and 4.2, this process is driven by itaconate-mediated succinate accumulation and HIF-1α stabilization (56), which is further amplified by a STING-dependent positive feedback loop that sustains M1 polarization (169). Notably, this metabolic-phenotypic coupling is further modulated by specific receptor pathways. In MASLD patients, hepatic DC-SIGN^+^ macrophages are significantly reduced. These macrophages directly bind to TLR4 and promote its endocytosis, which selectively suppresses MyD88-dependent pro-inflammatory signaling while enhancing TBK1-IRF3 activation, thereby alleviating liver disease (170). Integrative multi-omics analysis identified a DTNA^+^ macrophage subpopulation in the livers of patients with MASH. This subpopulation exhibits M2 polarization features, accompanied by enhanced glycolysis, increased TCA cycle activity, and upregulation of multiple pro-inflammatory pathways. It interacts with activated hepatic stellate cells (HSCs) via the RUNX2-PLG-PARD3 axis, thereby driving MASH progression and liver fibrosis (171). Spatially resolved multi-omics of 61 human liver samples (10 control, 17 MASLD, 34 MASH) has revealed that LAMs play a protective role in MASLD by clearing lipid droplets and secreting hepatocyte growth factor (HGF) (115). MITF was identified as a key regulator of the lipid-handling capacity of LAMs, and mass spectrometry imaging demonstrated MASLD-specific accumulation of phospholipids potentially linked to LAM-mediated phospholipid metabolism.

HSCs are the primary fibrogenic cells. Hepatocyte loss, inflammation, and metabolic alterations drive their transdifferentiation into myofibroblasts (172). Activated KCs, infiltrating macrophages, and injured hepatocytes secrete profibrogenic factors (e.g., PDGF, TGF-β1) that activate HSCs (173). Macrophages promote NF-κB activation in HSCs via IL-1 and TNF, enhancing myofibroblast survival and accelerating fibrosis (174). Phagocytosis of apoptotic hepatocytes by KCs upregulates death ligands (TNF-α, TRAIL, FasL), inducing further hepatocyte apoptosis and promoting inflammation and fibrosis (173, 175, 176). Residual cholesterol crystals in apoptotic hepatocytes activate the KC NLRP3 inflammasome (177). Foam-like KCs secrete chemokines to recruit monocytes/neutrophils and release TNF-α/TGF-β to activate HSCs, driving hepatic fibrosis (178, 179). Targeting macrophage scavenger receptor MSR1 with monoclonal antibodies prevents lipid-laden macrophage formation and inflammation in preclinical models (180).

Macrophage-derived grancalcin (GCA), which is upregulated in hepatic macrophages of MASH patients and correlates with disease severity, critically mediates macrophage-hepatocyte crosstalk. Mechanistically, GCA triggers TLR9-NF-κB signaling to promote pro-inflammatory cytokines, which induce hepatocyte lipid accumulation and apoptosis, and therapeutic GCA-blocking antibody effectively halts MASH progression (181). Macrophage MRC2 drives MASLD by binding CD147 to activate NF-κB and enhance TNF-α secretion, promoting hepatocyte lipid accumulation (182). As evidenced above, NAFLD progression results from multicellular interactions and dynamic regulation within the hepatic microenvironment. Collectively, understanding how metabolic reprogramming shapes macrophage phenotype—and how macrophage phenotype in turn reshapes tissue metabolism— is not only pathophysiologically critical but also therapeutically actionable.

### 5.3 Macrophages and pancreatic islet cells

**Figure 5:**
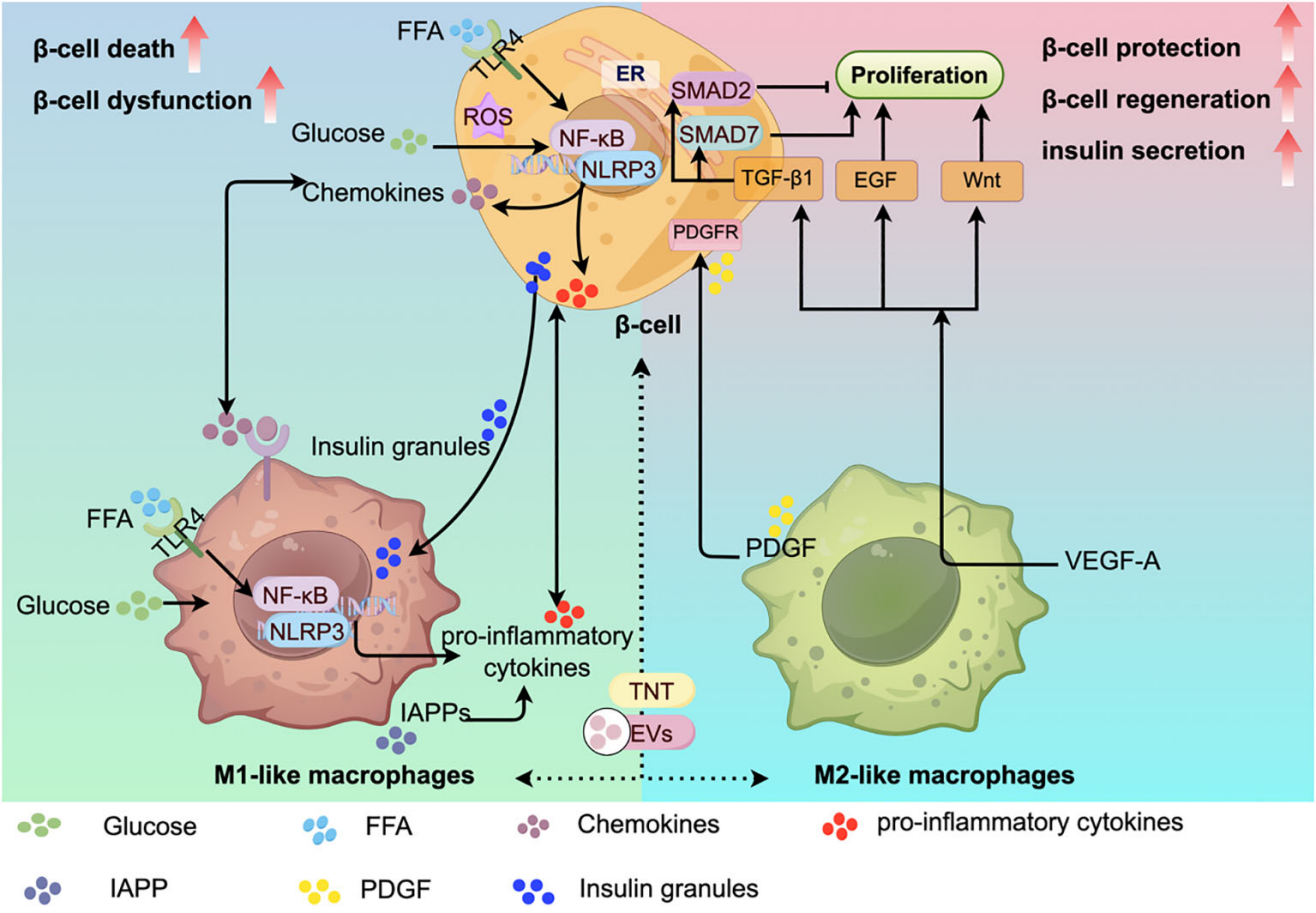
Interactions Between Pancreatic β-Cells and M1/M2 Macrophages. Under obese conditions, persistently elevated levels of blood glucose, FFAs, and IAPP promote macrophage polarization toward the M1 phenotype. The local expansion and accumulation of macrophages contribute to chronic islet inflammation, accompanied by the abundant production of various pro-inflammatory cytokines and chemokines. These factors activate inflammatory pathways such as NF-κB and NLRP3, leading to pancreatic β-cell dysfunction and even cell death, ultimately impairing GSIS. In contrast, M2 macrophages secrete various growth factors (e.g., TGF-β1, EGF, PDGF) that support β-cell protection and regeneration. Furthermore, macrophages engage in intercellular communication and material exchange with pancreatic β-cells via extracellular versicles (EVs) and tunneling nanotubes.

Islet-resident macrophages (IRMs) located adjacent to β-cells act as sensors of β-cell secretory capacity and viability. Their crosstalk with β−cells is central to islet inflammation in diabetes. In obesity−associated T2DM, continuously recruited macrophages drive chronic low−grade inflammation, promoting insulin resistance and β−cell dysfunction (183). Obesity induces localized expansion and accumulation of islet macrophages (184), mediated in part by PDGFR signaling, which contributes to β-cell proliferation defects and secretory dysfunction (185, 186). In T2DM, elevated free fatty acids (FFAs), ROS, and islet amyloid polypeptide (IAPP) promote M1-like macrophage polarization (**Figure 5****) (**187). Islet macrophages sense these metabolic cues and undergo local expansion (188). High glucose and FFAs induce pro-inflammatory cytokines (IL-1β, TNF-α, IL-6, CXCL1) in islets (189, 190). IL-1β, a key mediator, impairs β-cell function and induces dedifferentiation and apoptosis (187, 191). Palmitate activates the TLR4/NF-κB pathway in macrophages, recruiting M1 cells and impairing β-cell function (192). Phagocytosis of apoptotic β-cells by macrophages triggers efferocytosis-induced ROS and inflammasome activation, further propagating islet dysfunction (193).

IAPP, a peptide hormone synthesized and secreted by pancreatic β-cells, controls postprandial blood sugar levels by delaying gastric emptying and digestion, thereby reducing food intake. IAPP forms amyloid fibrils in T2DM, stimulating macrophage IL-1β secretion and impairing β-cell function (194); depletion of islet macrophages ameliorates this effect (195). Researchers have discovered that activation of free fatty acid receptor FFAR4 (GPR120) in islet macrophages mediates IL-6 release, thereby enhancing glucose-stimulated insulin secretion (GSIS) in lean mice. Yet in both obese type 2 diabetic humans and murine models, this FFAR4-mediated IL-6-dependent insulin secretion is impaired (196). Notably, exogenous IL-6 still improves GSIS in obese/diabetic islets, suggesting therapeutic potential. GPR92 is upregulated in islet macrophages of obese mice; its deficiency worsens glucose homeostasis and M1 polarization, while GPR92 activation promotes anti−inflammatory responses and improves β−cell function (197).

NLRP3 inflammasome activation in islet macrophages contributes to β−cell dysfunction; its deficiency preserves β−cell function in oxidative stress models (198, 199). Islet macrophages sense extracellular ATP (co−secreted with insulin) via purinergic signaling to monitor β−cell activity and maintain islet homeostasis (200, 201). STING activation in macrophages impairs GSIS by promoting phagocytosis of insulin secretory granules (202). M2-like macrophages support β-cell proliferation and survival. In injury models, recruited M2 macrophages promote β-cell regeneration via TGF-β1/SMAD7 and Wnt/β-catenin pathways (203–205).

Beyond phenotypic polarization, IRMs exhibit distinct metabolic features that shape their functional states. Single-cell RNA sequencing of human and mouse islets has revealed that IRMs possess an SLC7A11-driven antioxidant program, which maintains their survival while protecting β-cells from oxidative stress, thereby preserving insulin secretion (206). Conversely, under obese or diabetic conditions, islet macrophages undergo a glycolysis-dominant metabolic switch. In high-fat diet-induced diabetic mice, pancreatic macrophages show increased succinate accumulation and HIF-1α nuclear translocation, leading to enhanced production of pro-inflammatory cytokines and impaired β-cell insulin secretion; these effects can be reversed by the HIF-1α inhibitor PX478 (207). Thus, the succinate/HIF-1α axis represents a key metabolic checkpoint linking macrophage immunometabolism to β-cell dysfunction in T2DM.

Human data support the relevance of these metabolic states. In pancreatic tissues from T2DM patients, CD68^+^ macrophage density correlates positively with the severity of β-cell dysfunction (208). Single-cell transcriptomics of human T2DM islets (n=17) has identified distinct macrophage cytokine signatures (IL15, IFNα1, IFNβ, and IL17F) associated with islet inflammation and β-cell dysfunction (209). These findings underscore that islet macrophages are not a uniform population but rather a heterogeneous ensemble with diverse metabolic and functional states. The translational relevance of these metabolic insights is supported by pharmacological studies. The PPARγ agonist rosiglitazone has been shown to reduce macrophage-mediated β-cell death in type 1 diabetes models by downregulating macrophage heparanase expression, which enhances intra-islet extracellular matrix integrity and reduces immune cell infiltration (210).

Macrophages also communicate with β-cells through extracellular vesicles. Macrophage-derived exosomal miR-155 impairs β-cell function by targeting PDX1 (211), while M1-polarized macrophages transfer miR-212-5p to suppress insulin secretion via the SIRT2/Akt/GSK-3β/β-catenin pathway (212). Additionally, macrophages form tunneling nanotubes (TNTs) for intercellular transport of cytoplasmic material (213), though the relevance of this mechanism in islet biology remains to be explored. In summary, during obesity progression, pancreatic inflammation and β-cell dysfunction are closely associated with islet macrophages. Under homeostasis, macrophages maintain β-cell function, whereas under pathological conditions, they exacerbate β-cell damage through pro-inflammatory polarization, metabolic reprogramming, and intercellular communication.

## 6 Conclusion and perspectives

Macrophages are widely distributed across multiple tissues and organs throughout the body. By sensing microenvironmental signals and specific stimuli to polarize into pro-inflammatory and anti-inflammatory phenotypes, they coordinate metabolic adaptations and play a critical role in maintaining immune homeostasis. In the emerging field of immunometabolism, metabolic adaptation has been recognized as a key regulator of macrophage-mediated inflammation. Chronic low-grade inflammation contributes directly to obesity-related metabolic diseases—including insulin resistance, T2DM, and NAFLD—by modulating immunometabolic functions across tissues and organs. Therefore, understanding the metabolic characteristics of diverse macrophage populations and their regulatory mechanisms will facilitate the development of therapeutic strategies targeting macrophage metabolism.

Although no approved drugs directly target macrophage metabolism, several glucose-lowering, lipid-lowering, and anti-inflammatory agents in clinical use can indirectly reprogram macrophages, with more specific interventions already in clinical evaluation. Metformin, the first-line therapy for T2DM, inhibits mitochondrial complex I and shifts macrophage metabolism from glycolysis toward oxidative phosphorylation. Its anti-inflammatory effects on macrophages, mediated in part through AMPK activation, have been demonstrated in clinical studies using patient adipose tissue biopsies (214). Transcriptomic analyses of circulating immune cells from treated patients have revealed that SGLT2 inhibitors (dapagliflozin, empagliflozin) suppress M1 activation and promote M2 polarization via the NF-κB, AMPK/mTOR, and JAK/STAT pathways (215). These drugs exert multi-organ protective effects beyond glycemic control and represent promising candidates with immunomodulatory potential. In NASH, the dual CCR2/CCR5 antagonist cenicriviroc promotes M2-dominant polarization; although the Phase III AURORA trial missed its primary fibrosis endpoint, *post-hoc* analyses provided insights for subset-specific designs (216).

Beyond pharmacologic approaches, weight loss strategies such as caloric restriction and bariatric surgery consistently induce an M2-like shift in ATMs. The CALERIE−II trial demonstrated that caloric restriction suppresses adipocyte−derived SPARC, blocking NLRP3 inflammasome priming and JNK−mediated macrophage activation, thereby alleviating obesity−associated metabolic inflammation (217). Advances in nanomedicine enable precise macrophage reprogramming. Biomimetic liposomes and polymer-based nanoparticles have been engineered to achieve targeted delivery to inflammatory macrophages while minimizing off-target effects (218). In parallel, the incorporation of anti-phagocytic moieties such as CD47 on nanocarrier surfaces has been shown to reduce non-specific clearance by phagocytes, thereby improving targeted uptake (219). Beyond drug delivery, gene-therapy nanovesicles that generate cellular itaconate reservoirs have demonstrated the ability to reverse inflammation and restore metabolic function in T2D models by reprogramming macrophage glucose metabolism (220). Collectively, these findings highlight the therapeutic potential of targeting macrophage metabolism, though mechanism−based trials are still needed for clinical translation.

In this review, we have summarized the fundamental metabolic characteristics of macrophages and highlighted how they undergo metabolic rewiring to adapt to changing environmental cues. This underscores the importance of macrophage heterogeneity and plasticity in regulating cellular functions and shaping disease progression. Despite ongoing efforts, our understanding of the intricate regulatory networks within macrophages and between macrophages and their microenvironment remains limited, due to the broad tissue distribution of macrophages and the complexity of regulatory factors.

In obesity-related metabolic diseases, the emerging field of immunometabolism has directed attention toward the metabolic crosstalk between macrophages and target cells/organs (as discussed in Section 5). However, a substantial portion of current knowledge on macrophage metabolism derives from animal studies, and human data remain scarce. Future research should integrate single-cell sequencing, spatial transcriptomics, and high-dimensional multi-omics (proteogenomics, lipidomics, metabolomics) to dissect macrophage subpopulation heterogeneity and dynamic metabolic changes under obese conditions.

In summary, macrophages serve as crucial regulatory nodes in obesity-related metabolic diseases. Elucidating their metabolic reprogramming not only provides a new paradigm for understanding disease pathogenesis but also lays a vital foundation for developing novel therapeutic strategies.

## References

[1] González-Muniesa P Mártinez-González MA Hu FB Després JP Matsuzawa Y Loos RJF . Obesity. Nat Rev Dis Primers. (2017) 3:17034. doi: 10.1159/000177857 28617414

[2] NCD Risk Factor Collaboration (NCD-RisC) . Worldwide trends in underweight and obesity from 1990 to 2022: a pooled analysis of 3663 population-representative studies with 222 million children, adolescents, and adults. Lancet. (2024) 403:1027–50. doi: 10.1016/s0140-6736(23)02750-2 38432237 PMC7615769

[3] Jung UJ Choi MS . Obesity and its metabolic complications: the role of adipokines and the relationship between obesity, inflammation, insulin resistance, dyslipidemia and nonalcoholic fatty liver disease. Int J Mol Sci. (2014) 15:6184–223. doi: 10.3390/ijms15046184 24733068 PMC4013623

[4] Chew NWS Ng CH Tan DJH Kong G Lin C Chin YH . The global burden of metabolic disease: Data from 2000 to 2019. Cell Metab. (2023) 35:414–428.e3. doi: 10.1016/j.cmet.2023.02.003 36889281

[5] O'Neill LA Kishton RJ Rathmell J . A guide to immunometabolism for immunologists. Nat Rev Immunol. (2016) 16:553–65. doi: 10.1038/nri.2016.70 PMC5001910 27396447

[6] Sica A Mantovani A . Macrophage plasticity and polarization: *in vivo* veritas. J Clin Invest. (2012) 122:787–95. doi: 10.1172/jci59643 22378047 PMC3287223

[7] Luan H Horng T . Dynamic changes in macrophage metabolism modulate induction and suppression of Type I inflammatory responses. Curr Opin Immunol. (2021) 73:9–15. doi: 10.1016/j.coi.2021.07.012 34399114

[8] Gordon S Taylor PR . Monocyte and macrophage heterogeneity. Nat Rev Immunol. (2005) 5:953–64. doi: 10.1038/nri1733 16322748

[9] Tacke F . Targeting hepatic macrophages to treat liver diseases. J Hepatol. (2017) 66:1300–12. doi: 10.1016/j.jhep.2017.02.026 28267621

[10] Daemen S Schilling JD . The interplay between tissue niche and macrophage cellular metabolism in obesity. Front Immunol. (2019) 10:3133. doi: 10.3389/fimmu.2019.03133 32038642 PMC6987434

[11] Glass CK Natoli G . Molecular control of activation and priming in macrophages. Nat Immunol. (2016) 17:26–33. doi: 10.1038/ni.3306 26681459 PMC4795476

[12] Shapouri-Moghaddam A Mohammadian S Vazini H Taghadosi M Esmaeili SA Mardani F . Macrophage plasticity, polarization, and function in health and disease. J Cell Physiol. (2018) 233:6425–40. doi: 10.1002/jcp.26429 29319160 PMC13482560

[13] Appari M Channon KM McNeill E . Metabolic regulation of adipose tissue macrophage function in obesity and diabetes. Antioxid Redox Signal. (2018) 29:297–312. doi: 10.1089/ars.2017.7060 28661198 PMC6012981

[14] Vogel DY Glim JE Stavenuiter AW Breur M Heijnen P Amor S . Human macrophage polarization *in vitro*: maturation and activation methods compared. Immunobiology. (2014) 219:695–703. doi: 10.1016/j.imbio.2014.05.002 24916404

[15] Mosser DM Edwards JP . Exploring the full spectrum of macrophage activation. Nat Rev Immunol. (2008) 8:958–69. doi: 10.1038/nri2448 19029990 PMC2724991

[16] Orecchioni M Ghosheh Y Pramod AB Ley K . Macrophage polarization: Different gene signatures in M1(LPS+) vs. classically and M2(LPS-) vs. alternatively activated macrophages. Front Immunol. (2019) 10:1084. doi: 10.3389/fimmu.2019.01084 31178859 PMC6543837

[17] Sezginer O Unver N . Dissection of pro-tumoral macrophage subtypes and immunosuppressive cells participating in M2 polarization. Inflammation Res. (2024) 73:1411–23. doi: 10.1007/s00011-024-01907-3 38935134 PMC11349836

[18] Tang L Zhang H Wang C Li H Zhang Q Bai J . M2A and M2C macrophage subsets ameliorate inflammation and fibroproliferation in acute lung injury through interleukin 10 pathway. Shock. (2017) 48:119–29. doi: 10.1097/shk.0000000000000820 27941591

[19] Wang LX Zhang SX Wu HJ Rong XL Guo J . M2b macrophage polarization and its roles in diseases. J Leukoc Biol. (2019) 106:345–58. doi: 10.1002/jlb.3ru1018-378rr 30576000 PMC7379745

[20] Atri C Guerfali FZ Laouini D . Role of human macrophage polarization in inflammation during infectious diseases. Int J Mol Sci. (2018) 19:1801. doi: 10.3390/ijms19061801 29921749 PMC6032107

[21] Duluc D Delneste Y Tan F Moles MP Grimaud L Lenoir J . Tumor-associated leukemia inhibitory factor and IL-6 skew monocyte differentiation into tumor-associated macrophage-like cells. Blood. (2007) 110:4319–30. doi: 10.1182/blood-2007-02-072587 17848619

[22] Wu H Xu JB He YL Peng JJ Zhang XH Chen CQ . Tumor-associated macrophages promote angiogenesis and lymphangiogenesis of gastric cancer. J Surg Oncol. (2012) 106:462–8. doi: 10.1002/jso.23110 22488237

[23] Vats D Mukundan L Odegaard JI Zhang L Smith KL Morel CR . Oxidative metabolism and PGC-1beta attenuate macrophage-mediated inflammation. Cell Metab. (2006) 4:13–24. doi: 10.1016/j.cmet.2006.05.011 16814729 PMC1904486

[24] Freemerman AJ Johnson AR Sacks GN Milner JJ Kirk EL Troester MA . Metabolic reprogramming of macrophages: glucose transporter 1 (GLUT1)-mediated glucose metabolism drives a proinflammatory phenotype. J Biol Chem. (2014) 289:7884–96. doi: 10.1074/jbc.M113.522037 PMC3953299 24492615

[25] Aktan F . iNOS-mediated nitric oxide production and its regulation. Life Sci. (2004) 75:639–53. doi: 10.1016/j.lfs.2003.10.042 15172174

[26] Saha S Shalova IN Biswas SK . Metabolic regulation of macrophage phenotype and function. Immunol Rev. (2017) 280:102–11. doi: 10.1111/imr.12603 29027220

[27] Sowers ML Tang H Singh VK Khan A Mishra A Restrepo BI . Multi-OMICs analysis reveals metabolic and epigenetic changes associated with macrophage polarization. J Biol Chem. (2022) 298:102418. doi: 10.1016/j.jbc.2022.102418 36030823 PMC9525912

[28] Russo S Kwiatkowski M Govorukhina N Bischoff R Melgert BN . Meta-inflammation and metabolic reprogramming of macrophages in diabetes and obesity: the importance of metabolites. Front Immunol. (2021) 12:746151. doi: 10.3389/fimmu.2021.746151 34804028 PMC8602812

[29] Warburg O Wind F Negelein E . The metabolism of tumors in the body. J Gen Physiol. (1927) 8:519–30. doi: 10.1085/jgp.8.6.519 19872213 PMC2140820

[30] Mathis D Shoelson SE . Immunometabolism: an emerging frontier. Nat Rev Immunol. (2011) 11:81. doi: 10.1038/nri2922 21469396 PMC4784680

[31] Van Den Bossche J O'Neill LA Menon D . Macrophage immunometabolism: Where are we (going)? Trends Immunol. (2017) 38:395–406. doi: 10.1016/j.it.2017.03.001 28396078

[32] Zhu L Zhao Q Yang T Ding W Zhao Y . Cellular metabolism and macrophage functional polarization. Int Rev Immunol. (2015) 34:82–100. doi: 10.3109/08830185.2014.969421 25340307

[33] Van den Bossche J Baardman J Otto NA van der Velden S Neele AE van den Berg SM . Mitochondrial dysfunction prevents repolarization of inflammatory macrophages. Cell Rep. (2016) 17:684–96. doi: 10.1016/j.celrep.2016.09.008 27732846

[34] Lunt SY Vander Heiden MG . Aerobic glycolysis: meeting the metabolic requirements of cell proliferation. Annu Rev Cell Dev Biol. (2011) 27:441–64. doi: 10.1146/annurev-cellbio-092910-154237 21985671

[35] Fukuzumi M Shinomiya H Shimizu Y Ohishi K Utsumi S . Endotoxin-induced enhancement of glucose influx into murine peritoneal macrophages via GLUT1. Infect Immun. (1996) 64:108–12. doi: 10.1128/iai.64.1.108-112.1996 8557327 PMC173734

[36] Nishizawa T Kanter JE Kramer F Barnhart S Shen X Vivekanandan-Giri A . Testing the role of myeloid cell glucose flux in inflammation and atherosclerosis. Cell Rep. (2014) 7:356–65. doi: 10.1016/j.celrep.2014.03.028 24726364 PMC4021396

[37] Wang T Liu H Lian G Zhang SY Wang X Jiang C . HIF1α-induced glycolysis metabolism is essential to the activation of inflammatory macrophages. Mediators Inflammation. (2017) 2017:9029327. doi: 10.1155/2017/9029327 29386753 PMC5745720

[38] Cramer T Yamanishi Y Clausen BE Förster I Pawlinski R Mackman N . HIF-1alpha is essential for myeloid cell-mediated inflammation. Cell. (2003) 112:645–57. doi: 10.1016/s0092-8674(03)00154-5 12628185 PMC4480774

[39] Rius J Guma M Schachtrup C Akassoglou K Zinkernagel AS Nizet V . NF-kappaB links innate immunity to the hypoxic response through transcriptional regulation of HIF-1alpha. Nature. (2008) 453:807–11. doi: 10.1038/nature06905 18432192 PMC2669289

[40] Koivunen P Hirsilä M Remes AM Hassinen IE Kivirikko KI Myllyharju J . Inhibition of hypoxia-inducible factor (HIF) hydroxylases by citric acid cycle intermediates: possible links between cell metabolism and stabilization of HIF. J Biol Chem. (2007) 282:4524–32. doi: 10.1074/jbc.m610415200 17182618

[41] Kaplon J Zheng L Meissl K Chaneton B Selivanov VA Mackay G . A key role for mitochondrial gatekeeper pyruvate dehydrogenase in oncogene-induced senescence. Nature. (2013) 498:109–12. doi: 10.1038/nature12154 23685455

[42] Kumar M Sharma S Kumar J Barik S Mazumder S . Mitochondrial electron transport chain in macrophage reprogramming: Potential role in antibacterial immune response. Curr Res Immunol. (2024) 5:100077. doi: 10.1016/j.crimmu.2024.100077 38572399 PMC10987323

[43] Kim JW Tchernyshyov I Semenza GL Dang CV . HIF-1-mediated expression of pyruvate dehydrogenase kinase: a metabolic switch required for cellular adaptation to hypoxia. Cell Metab. (2006) 3:177–85. doi: 10.1016/j.cmet.2006.02.002 16517405

[44] Semba H Takeda N Isagawa T Sugiura Y Honda K Wake M . HIF-1α-PDK1 axis-induced active glycolysis plays an essential role in macrophage migratory capacity. Nat Commun. (2016) 7:11635. doi: 10.1038/ncomms11635 27189088 PMC4873978

[45] Huang SC Smith AM Everts B Colonna M Pearce EL Schilling JD . Metabolic reprogramming mediated by the mTORC2-IRF4 signaling axis is essential for macrophage alternative activation. Immunity. (2016) 45:817–30. doi: 10.1016/j.immuni.2016.09.016 27760338 PMC5535820

[46] Covarrubias AJ AksoylAr HI Horng T . Control of macrophage metabolism and activation by mTOR and Akt signaling. Semin Immunol. (2015) 27:286–96. doi: 10.1016/j.smim.2015.08.001 26360589 PMC4682888

[47] Tan Z Xie N Cui H Moellering DR Abraham E Thannickal VJ . Pyruvate dehydrogenase kinase 1 participates in macrophage polarization via regulating glucose metabolism. J Immunol. (2015) 194:6082–9. doi: 10.4049/jimmunol.1402469 25964487 PMC4458459

[48] Wang F Zhang S Vuckovic I Jeon R Lerman A Folmes CD . Glycolytic stimulation is not a requirement for M2 macrophage differentiation. Cell Metab. (2018) 28:463–475.e4. doi: 10.1016/j.cmet.2018.08.012 30184486 PMC6449248

[49] Wculek SK Heras-Murillo I Mastrangelo A Mañanes D Galán M Miguel V . Oxidative phosphorylation selectively orchestrates tissue macrophage homeostasis. Immunity. (2023) 56:516–530.e9. doi: 10.1016/j.immuni.2023.01.011 36738738

[50] Jha AK Huang SC Sergushichev A Lampropoulou V Ivanova Y Loginicheva E . Network integration of parallel metabolic and transcriptional data reveals metabolic modules that regulate macrophage polarization. Immunity. (2015) 42:419–30. doi: 10.1016/j.immuni.2015.02.005 25786174

[51] Kelly B O'Neill LA . Metabolic reprogramming in macrophages and dendritic cells in innate immunity. Cell Res. (2015) 25:771–84. doi: 10.1038/cr.2015.68 26045163 PMC4493277

[52] Infantino V Iacobazzi V Palmieri F Menga A . ATP-citrate lyase is essential for macrophage inflammatory response. Biochem Biophys Res Commun. (2013) 440:105–11. doi: 10.1016/j.bbrc.2013.09.037 24051091

[53] O'Neill LAJ Artyomov MN . Itaconate: the poster child of metabolic reprogramming in macrophage function. Nat Rev Immunol. (2019) 19:273–81. doi: 10.1038/s41577-019-0128-5 30705422

[54] Tannahill GM Curtis AM Adamik J Palsson-McDermott EM McGettrick AF Goel G . Succinate is an inflammatory signal that induces IL-1β through HIF-1α. Nature. (2013) 496:238–42. doi: 10.1038/nature11986 PMC4031686 23535595

[55] Palsson-McDermott EM Curtis AM Goel G Lauterbach MAR Sheedy FJ Gleeson LE . Pyruvate kinase M2 regulates Hif-1α activity and IL-1β induction and is a critical determinant of the Warburg effect in LPS-activated macrophages. Cell Metab. (2015) 21:347. doi: 10.1016/j.cmet.2014.12.005 29510100

[56] Lampropoulou V Sergushichev A Bambouskova M Nair S Vincent EE Loginicheva E . Itaconate links inhibition of succinate dehydrogenase with macrophage metabolic remodeling and regulation of inflammation. Cell Metab. (2016) 24:158–66. doi: 10.1016/j.cmet.2016.06.004 27374498 PMC5108454

[57] He W Miao FJ Lin DC Schwandner RT Wang Z Gao J . Citric acid cycle intermediates as ligands for orphan G-protein-coupled receptors. Nature. (2004) 429:188–93. doi: 10.1038/nature02488 15141213

[58] Littlewood-Evans A Sarret S Apfel V Loesle P Dawson J Zhang J . GPR91 senses extracellular succinate released from inflammatory macrophages and exacerbates rheumatoid arthritis. J Exp Med. (2016) 213:1655–62. doi: 10.1084/jem.20160061 27481132 PMC4995082

[59] Liu PS Wang H Li X Chao T Teav T Christen S . α-ketoglutarate orchestrates macrophage activation through metabolic and epigenetic reprogramming. Nat Immunol. (2017) 18:985–94. doi: 10.1038/ni.3796 28714978

[60] Palmieri EM Menga A Martín-Pérez R Quinto A Riera-Domingo C De Tullio G . Pharmacologic or genetic targeting of glutamine synthetase skews macrophages toward an M1-like phenotype and inhibits tumor metastasis. Cell Rep. (2017) 20:1654–66. doi: 10.1016/j.celrep.2017.07.054 28813676 PMC5575233

[61] Mills EL Kelly B Logan A Costa ASH Varma M Bryant CE . Succinate dehydrogenase supports metabolic repurposing of mitochondria to drive inflammatory macrophages. Cell. (2016) 167:457–70.e13. doi: 10.1016/j.cell.2016.08.064 27667687 PMC5863951

[62] Wang Y Li N Zhang X Horng T . Mitochondrial metabolism regulates macrophage biology. J Biol Chem. (2021) 297:100904. doi: 10.1016/j.jbc.2021.100904 34157289 PMC8294576

[63] Forrester S J Kikuchi D S Hernandes M S . Reactive oxygen species in metabolic and inflammatory signaling. Circ Res. (2018) 122(6):877–902. 10.1161/CIRCRESAHA.117.311401 PMC5926825 29700084

[64] Fuhrmann DC Wittig I Brüne B . TMEM126B deficiency reduces mitochondrial SDH oxidation by LPS, attenuating HIF-1α stabilization and IL-1β expression. Redox Biol. (2019) 20:204–16. doi: 10.1016/j.redox.2018.10.007 30368040 PMC6202876

[65] Schleh MW Caslin HL Garcia JN Mashayekhi M Srivastava G Bradley AB . Metaflammation in obesity and its therapeutic targeting. Sci Transl Med. (2023) 15:eadf9382. doi: 10.1126/scitranslmed.adf9382 37992150 PMC10847980

[66] Purvis GSD Collino M van Dam AD Einaudi G Ng Y Shanmuganathan M . OxPhos in adipose tissue macrophages regulated by BTK enhances their M2-like phenotype and confers a systemic immunometabolic benefit in obesity. Diabetes. (2024). doi: 10.1101/2023.10.09.561199 38193882

[67] Yin M O'Neill LAJ . The role of the electron transport chain in immunity. FASEB J. (2021) 35:e21974. doi: 10.1096/fj.202101161r 34793601

[68] Moon JS da Cunha FF Huh JY Andreyev AY Lee J Mahata SK . ANT2 drives proinflammatory macrophage activation in obesity. JCI Insight. (2021) 6:e147033. doi: 10.1172/jci.insight.147033 34676827 PMC8564915

[69] Newsholme EA Newsholme P Curi R . The role of the citric acid cycle in cells of the immune system and its importance in sepsis, trauma and burns. Biochem Soc Symp. (1987) 54:145–62. 3332991

[70] Nagy C Haschemi A . Time and demand are two critical dimensions of immunometabolism: the process of macrophage activation and the pentose phosphate pathway. Front Immunol. (2015) 6:164. doi: 10.3389/fimmu.2015.00164 25904920 PMC4389563

[71] Ray PD Huang BW Tsuji Y . Reactive oxygen species (ROS) homeostasis and redox regulation in cellular signaling. Cell Signal. (2012) 24:981–90. doi: 10.1016/j.cellsig.2012.01.008 22286106 PMC3454471

[72] Sun L Zhou F Shao Y Lv Z Li C . Sedoheptulose kinase bridges the pentose phosphate pathway and immune responses in pathogen-challenged sea cucumber Apostichopus japonicus. Dev Comp Immunol. (2020) 109:103694. doi: 10.1016/j.dci.2020.103694 32283109

[73] Park YJ Choe SS Sohn JH Kim JB . The role of glucose-6-phosphate dehydrogenase in adipose tissue inflammation in obesity. Adipocyte. (2017) 6:147–53. doi: 10.1080/21623945.2017.1288321 28425844 PMC5477698

[74] Ham M Choe SS Shin KC Choi G Kim JW Noh JR . Glucose-6-phosphate dehydrogenase deficiency improves insulin resistance with reduced adipose tissue inflammation in obesity. Diabetes. (2016) 65:2624–38. doi: 10.2337/db16-0060 27284106

[75] Ham M Lee JW Choi AH Jang H Choi G Park J . Macrophage glucose-6-phosphate dehydrogenase stimulates proinflammatory responses with oxidative stress. Mol Cell Biol. (2013) 33:2425–35. doi: 10.1128/mcb.01260-12 23572562 PMC3700093

[76] Haschemi A Kosma P Gille L Evans CR Burant CF Starkl P . The sedoheptulose kinase CARKL directs macrophage polarization through control of glucose metabolism. Cell Metab. (2012) 15:813–26. doi: 10.1016/j.cmet.2012.04.023 22682222 PMC3370649

[77] Yan J Horng T . Lipid metabolism in regulation of macrophage functions. Trends Cell Biol. (2020) 30:979–89. doi: 10.1016/j.tcb.2020.09.006 33036870

[78] Feingold KR Shigenaga JK Kazemi MR McDonald CM Patzek SM Cross AS . Mechanisms of triglyceride accumulation in activated macrophages. J Leukoc Biol. (2012) 92:829–39. doi: 10.1189/jlb.1111537 22753953 PMC3441312

[79] Endemann G Stanton LW Madden KS Bryant CM White RT Protter AA . CD36 is a receptor for oxidized low density lipoprotein. J Biol Chem. (1993) 268:11811–6. doi: 10.1016/s0021-9258(19)50272-1 7685021

[80] Wu Q Ortegon AM Tsang B Doege H Feingold KR Stahl A . FATP1 is an insulin-sensitive fatty acid transporter involved in diet-induced obesity. Mol Cell Biol. (2006) 26:3455–67. doi: 10.1128/mcb.26.9.3455-3467.2006 16611988 PMC1447434

[81] Ecker J Liebisch G Englmaier M Grandl M Robenek H Schmitz G . Induction of fatty acid synthesis is a key requirement for phagocytic differentiation of human monocytes. Proc Natl Acad Sci USA. (2010) 107:7817–22. doi: 10.1073/pnas.0912059107 20385828 PMC2867858

[82] Im SS Yousef L Blaschitz C Liu JZ Edwards RA Young SG . Linking lipid metabolism to the innate immune response in macrophages through sterol regulatory element binding protein-1a. Cell Metab. (2011) 13:540–9. doi: 10.1016/j.cmet.2011.04.001 21531336 PMC3090630

[83] Carroll RG Zasłona Z Galván-Peña S Koppe EL Sévin DC Angiari S . An unexpected link between fatty acid synthase and cholesterol synthesis in proinflammatory macrophage activation. J Biol Chem. (2018) 293:5509–21. doi: 10.1074/jbc.ra118.001921 29463677 PMC5900750

[84] Wei X Song H Yin L Rizzo MG Sidhu R Covey DF . Fatty acid synthesis configures the plasma membrane for inflammation in diabetes. Nature. (2016) 539:294–8. doi: 10.1038/nature20117 27806377 PMC5671339

[85] Joseph SB Bradley MN Castrillo A Bruhn KW Mak PA Pei L . LXR-dependent gene expression is important for macrophage survival and the innate immune response. Cell. (2004) 119:299–309. doi: 10.1016/j.cell.2004.09.032 15479645

[86] Qian X Yang Z Mao E Chen E . Regulation of fatty acid synthesis in immune cells. Scand J Immunol. (2018) 88:e12713. doi: 10.1111/sji.12713 30176060

[87] Hong C Walczak R Dhamko H Bradley MN Marathe C Boyadjian R . Constitutive activation of LXR in macrophages regulates metabolic and inflammatory gene expression: identification of ARL7 as a direct target. J Lipid Res. (2011) 52:531–9. doi: 10.1194/jlr.m010686 21187453 PMC3035689

[88] Huang SC Everts B Ivanova Y O'Sullivan D Nascimento M Smith AM . Cell-intrinsic lysosomal lipolysis is essential for alternative activation of macrophages. Nat Immunol. (2014) 15:846–55. doi: 10.1038/ni.2956 25086775 PMC4139419

[89] Mills EL O'Neill LA . Reprogramming mitochondrial metabolism in macrophages as an anti-inflammatory signal. Eur J Immunol. (2016) 46:13–21. doi: 10.1002/eji.201445427 26643360

[90] Malandrino MI Fucho R Weber M Calderon-Dominguez M Mir JF Valcarcel L . Enhanced fatty acid oxidation in adipocytes and macrophages reduces lipid-induced triglyceride accumulation and inflammation. Am J Physiol Endocrinol Metab. (2015) 308:E756–69. doi: 10.1152/ajpendo.00362.2014 25714670

[91] Odegaard JI Ricardo-Gonzalez RR Goforth MH Morel CR Subramanian V Mukundan L . Macrophage-specific PPARgamma controls alternative activation and improves insulin resistance. Nature. (2007) 447:1116–20. doi: 10.1038/nature05894 17515919 PMC2587297

[92] Nelson VL Nguyen HCB Garcìa-Cañaveras JC Briggs ER Ho WY DiSpirito JR . PPARγ is a nexus controlling alternative activation of macrophages via glutamine metabolism. Genes Dev. (2018) 32:1035–44. doi: 10.1101/gad.312355.118 30006480 PMC6075146

[93] Divakaruni AS Hsieh WY Minarrieta L Duong TN Kim KKO Desousa BR . Etomoxir inhibits macrophage polarization by disrupting CoA homeostasis. Cell Metab. (2018) 28:490–503.e7. doi: 10.1016/j.cmet.2018.06.001 30043752 PMC6125190

[94] Nomura M Liu J Rovira II Gonzalez-Hurtado E Lee J Wolfgang MJ . Fatty acid oxidation in macrophage polarization. Nat Immunol. (2016) 17:216–7. doi: 10.1038/ni.3366 26882249 PMC6033271

[95] Namgaladze D Brüne B . Fatty acid oxidation is dispensable for human macrophage IL-4-induced polarization. Biochim Biophys Acta. (2014) 1841:1329–35. doi: 10.1016/j.bbalip.2014.06.007 24960101

[96] Applegate CC Kang Y Deng H Chen D Gonzalez Medina NY Cui Y . Nanomedicine targeting PPAR in adipose tissue macrophages improves lipid metabolism and obesity-induced metabolic dysfunction. Sci Adv. (2025) 11:eads3731. doi: 10.1126/sciadv.ads3731 41004576 PMC12466850

[97] Zimmermann JA Lucht K Stecher M Badhan C Glaser KM Epple MW . Functional multi-organelle units control inflammatory lipid metabolism of macrophages. Nat Cell Biol. (2024) 26:1261–73. doi: 10.1038/s41556-024-01457-0 38969763 PMC11321999

[98] Li L Meng Y Li Z Dai W Xu X Bi X . Discovery and development of small molecule modulators targeting glutamine metabolism. Eur J Med Chem. (2019) 163:215–42. doi: 10.1016/j.ejmech.2018.11.066 30522056

[99] Liu PS Ho PC . Determining macrophage polarization upon metabolic perturbation. Methods Mol Biol. (2019) 1862:173–86. doi: 10.1007/978-1-4939-8769-6_13 30315468

[100] Rath M Müller I Kropf P Closs EI Munder M . Metabolism via arginase or nitric oxide synthase: two competing arginine pathways in macrophages. Front Immunol. (2014) 5:532. doi: 10.3389/fimmu.2014.00532 25386178 PMC4209874

[101] Chawla A . Control of macrophage activation and function by PPARs. Circ Res. (2010) 106:1559–69. doi: 10.1161/circresaha.110.216523 20508200 PMC2897247

[102] Nguyen KD Qiu Y Cui X Goh YP Mwangi J David T . Alternatively activated macrophages produce catecholamines to sustain adaptive thermogenesis. Nature. (2011) 480:104–8. doi: 10.1038/nature10653 22101429 PMC3371761

[103] Das P Lahiri A Lahiri A Chakravortty D . Modulation of the arginase pathway in the context of microbial pathogenesis: a metabolic enzyme moonlighting as an immune modulator. PLoS Pathog. (2010) 6:e1000899. doi: 10.1371/journal.ppat.1000899 20585552 PMC2887468

[104] McGaha TL Huang L Lemos H Metz R Mautino M Prendergast GC . Amino acid catabolism: a pivotal regulator of innate and adaptive immunity. Immunol Rev. (2012) 249:135–57. doi: 10.1111/j.1600-065x.2012.01149.x 22889220 PMC4384693

[105] Platten M Wick W Van Den Eynde BJ . Tryptophan catabolism in cancer: beyond IDO and tryptophan depletion. Cancer Res. (2012) 72:5435–40. doi: 10.1158/0008-5472.can-12-0569 23090118

[106] Wang XF Wang HS Wang H Zhang F Wang KF Guo Q . The role of indoleamine 2,3-dioxygenase (IDO) in immune tolerance: focus on macrophage polarization of THP-1 cells. Cell Immunol. (2014) 289:42–8. doi: 10.1016/j.cellimm.2014.02.005 24721110

[107] Kaiser H Parker E Hamrick MW . Kynurenine signaling through the aryl hydrocarbon receptor: Implications for aging and healthspan. Exp Gerontol. (2020) 130:110797. doi: 10.1016/j.exger.2019.110797 31786316 PMC7899131

[108] Stephens GL Wang Q Swerdlow B Bhat G Kolbeck R Fung M . Kynurenine 3-monooxygenase mediates inhibition of Th17 differentiation via catabolism of endogenous aryl hydrocarbon receptor ligands. Eur J Immunol. (2013) 43:1727–34. doi: 10.1002/eji.201242779 23568529

[109] Yue Y Huang W Liang J Guo J Ji J Yao Y . IL4I1 is a novel regulator of M2 macrophage polarization that can inhibit T cell activation via L-tryptophan and arginine depletion and IL-10 production. PLoS One. (2015) 10:e0142979. doi: 10.1371/journal.pone.0142979 26599209 PMC4658051

[110] Olefsky JM Glass CK . Macrophages, inflammation, and insulin resistance. Annu Rev Physiol. (2010) 72:219–46. doi: 10.1146/annurev-physiol-021909-135846 20148674

[111] Wu H Ballantyne CM . Metabolic inflammation and insulin resistance in obesity. Circ Res. (2020) 126:1549–64. doi: 10.1161/circresaha.119.315896 32437299 PMC7250139

[112] Peterson KR Cottam MA Kennedy AJ Hasty AH . Macrophage-targeted therapeutics for metabolic disease. Trends Pharmacol Sci. (2018) 39:536–46. doi: 10.1016/j.tips.2018.03.001 29628274 PMC5962426

[113] Grosjean A Venteclef N Dalmas E . Understanding the heterogeneity and functions of metabolic tissue macrophages. Semin Cell Dev Biol. (2021) 119:130–9. doi: 10.1016/j.semcdb.2021.09.002 34561168

[114] Jaitin DA Adlung L Thaiss CA Weiner A Li B Descamps H . Lipid-associated macrophages control metabolic homeostasis in a Trem2-dependent manner. Cell. (2019) 178:686–98.e14. doi: 10.1016/j.cell.2019.05.054 31257031 PMC7068689

[115] Li Z Luo G Gan C Zhang H Li L Zhang X . Spatially resolved multi-omics of human metabolic dysfunction-associated steatotic liver disease. Nat Genet. (2025) 57:3112–25. doi: 10.1038/s41588-025-02407-8 41286103 PMC12695644

[116] Bhatia K Tiwari S Gupta VK Sapariya NM Upadhyay SK . An *in vitro* model of adipose tissue-associated macrophages. J Biosci. (2024) 49:79. doi: 10.1007/s12038-024-00464-5 39119914

[117] Kratz M Coats BR Hisert KB Hagman D Mutskov V Peris E . Metabolic dysfunction drives a mechanistically distinct proinflammatory phenotype in adipose tissue macrophages. Cell Metab. (2014) 20:614–25. doi: 10.1016/j.cmet.2014.08.010 25242226 PMC4192131

[118] Vasamsetti SB Sadaf S Uddin MA Shen J Johny E Mondal A . Tissue-resident macrophage survival depends on mitochondrial function regulated by SerpinB2 in chronic inflammation. Nat Commun. (2026) 17:1493. doi: 10.1038/s41467-026-69196-4 41680163 PMC12902017

[119] Weisberg SP McCann D Desai M Rosenbaum M Leibel RL Ferrante Jr AW . Obesity is associated with macrophage accumulation in adipose tissue. J Clin Invest. (2003) 112:1796–808. doi: 10.1172/jci200319246 14679176 PMC296995

[120] Catrysse L Van Loo G . Adipose tissue macrophages and their polarization in health and obesity. Cell Immunol. (2018) 330:114–9. doi: 10.1016/j.cellimm.2018.03.001 29526353

[121] Lumeng CN Bodzin JL Saltiel AR . Obesity induces a phenotypic switch in adipose tissue macrophage polarization. J Clin Invest. (2007) 117:175–84. doi: 10.1172/jci29881 17200717 PMC1716210

[122] Strissel KJ Stancheva Z Miyoshi H Perfield JW DeFuria J Jick Z . Adipocyte death, adipose tissue remodeling, and obesity complications. Diabetes. (2007) 56:2910–8. doi: 10.2337/db07-0767 17848624

[123] Xu X Grijalva A Skowronski A van Eijk M Serlie MJ Ferrante Jr AW . Obesity activates a program of lysosomal-dependent lipid metabolism in adipose tissue macrophages independently of classic activation. Cell Metab. (2013) 18:816–30. doi: 10.1016/j.cmet.2013.11.001 24315368 PMC3939841

[124] Bremer AA Devaraj S Afify A Jialal I . Adipose tissue dysregulation in patients with metabolic syndrome. J Clin Endocrinol Metab. (2011) 96:E1782–8. doi: 10.1210/jc.2011-1577 21865369 PMC3205887

[125] Mouton AJ Li X Hall ME Hall JE . Obesity, hypertension, and cardiac dysfunction: novel roles of immunometabolism in macrophage activation and inflammation. Circ Res. (2020) 126:789–806. doi: 10.1161/CIRCRESAHA.119.312321 32163341 PMC7255054

[126] Aguirre V Uchida T Yenush L Davis R White MF . The c-Jun NH(2)-terminal kinase promotes insulin resistance during association with insulin receptor substrate-1 and phosphorylation of Ser(307). J Biol Chem. (2000) 275:9047–56. doi: 10.1074/jbc.275.12.9047 10722755

[127] Gual P Le Marchand-Brustel Y Tanti JF . Positive and negative regulation of insulin signaling through IRS-1 phosphorylation. Biochimie. (2005) 87:99–109. doi: 10.1016/j.biochi.2004.10.019 15733744

[128] Jiao P Chen Q Shah S Du J Tao B Tzameli I . Obesity-related upregulation of monocyte chemotactic factors in adipocytes: involvement of nuclear factor-kappaB and c-Jun NH2-terminal kinase pathways. Diabetes. (2009) 58:104–15. doi: 10.2337/db07-1344 18835938 PMC2606857

[129] Nguyen MT Favelyukis S Nguyen AK Reichart D Scott PA Jenn A . A subpopulation of macrophages infiltrates hypertrophic adipose tissue and is activated by free fatty acids via Toll-like receptors 2 and 4 and JNK-dependent pathways. J Biol Chem. (2007) 282:35279–92. doi: 10.1074/jbc.m706762200 17916553

[130] Wen H Gris D Lei Y Jha S Zhang L Huang MT . Fatty acid-induced NLRP3-ASC inflammasome activation interferes with insulin signaling. Nat Immunol. (2011) 12:408–15. doi: 10.1038/ni.2022 21478880 PMC4090391

[131] Shi H Kokoeva MV Inouye K Tzameli I Yin H Flier JS . TLR4 links innate immunity and fatty acid-induced insulin resistance. J Clin Invest. (2006) 116:3015–25. doi: 10.1172/jci28898 17053832 PMC1616196

[132] Suganami T Nishida J Ogawa Y . A paracrine loop between adipocytes and macrophages aggravates inflammatory changes: role of free fatty acids and tumor necrosis factor alpha. Arterioscler Thromb Vasc Biol. (2005) 25:2062–8. doi: 10.1161/01.atv.0000183883.72263.13 16123319

[133] Hotamisligil GS . Inflammation, metaflammation and immunometabolic disorders. Nature. (2017) 542:177–85. doi: 10.1038/nature21363 28179656

[134] Guilherme A Virbasius JV Puri V Czech MP . Adipocyte dysfunctions linking obesity to insulin resistance and type 2 diabetes. Nat Rev Mol Cell Biol. (2008) 9:367–77. doi: 10.1038/nrm2391 18401346 PMC2886982

[135] Lancaster GI Langley KG Berglund NA Kammoun HL Reibe S Estevez E . Evidence that TLR4 is not a receptor for saturated fatty acids but mediates lipid-induced inflammation by reprogramming macrophage metabolism. Cell Metab. (2018) 27:1096–110.e5. doi: 10.1016/j.cmet.2018.03.014 29681442

[136] Ohashi K Parker JL Ouchi N Higuchi A Vita JA Gokce N . Adiponectin promotes macrophage polarization toward an anti-inflammatory phenotype. J Biol Chem. (2010) 285:6153–60. doi: 10.1074/jbc.m109.088708 20028977 PMC2825410

[137] Luo Y Liu M . Adiponectin: a versatile player of innate immunity. J Mol Cell Biol. (2016) 8:120–8. doi: 10.1093/jmcb/mjw012 26993045 PMC4816149

[138] Okla M Zaher W Alfayez M Chung S . Inhibitory effects of Toll-like receptor 4, NLRP3 inflammasome, and interleukin-1β on white adipocyte browning. Inflammation. (2018) 41:626–42. doi: 10.1007/s10753-017-0718-y 29264745 PMC6066287

[139] Evans RM Barish GD Wang YX . PPARs and the complex journey to obesity. Nat Med. (2004) 10:355–61. doi: 10.2302/kjm.53.53 15057233

[140] Buczynski MW Stephens DL Bowers-Gentry RC Grkovich A Deems RA Dennis EA . TLR-4 and sustained calcium agonists synergistically produce eicosanoids independent of protein synthesis in RAW264.7 cells. J Biol Chem. (2007) 282:22834–47. doi: 10.1074/jbc.m701831200 17535806

[141] Silverstein RL Febbraio M . CD36, a scavenger receptor involved in immunity, metabolism, angiogenesis, and behavior. Sci Signal. (2009) 2:re3. doi: 10.1126/scisignal.272re3 19471024 PMC2811062

[142] Oh DY Talukdar S Bae EJ Imamura T Morinaga H Fan W . GPR120 is an omega-3 fatty acid receptor mediating potent anti-inflammatory and insulin-sensitizing effects. Cell. (2010) 142:687–98. doi: 10.1016/j.cell.2010.07.041 20813258 PMC2956412

[143] Yeop Han C Kargi AY Omer M Chan CK Wabitsch M O'Brien KD . Differential effect of saturated and unsaturated free fatty acids on the generation of monocyte adhesion and chemotactic factors by adipocytes: dissociation of adipocyte hypertrophy from inflammation. Diabetes. (2010) 59:386–96. doi: 10.2337/db09-0925 19934003 PMC2809975

[144] Odegaard JI Ricardo-Gonzalez RR Red Eagle A Vats D Morel CR Goforth MH . Alternative M2 activation of Kupffer cells by PPARdelta ameliorates obesity-induced insulin resistance. Cell Metab. (2008) 7:496–507. doi: 10.1016/j.cmet.2008.04.003 18522831 PMC2587370

[145] Kang K Reilly SM Karabacak V Gangl MR Fitzgerald K Hatano B . Adipocyte-derived Th2 cytokines and myeloid PPARdelta regulate macrophage polarization and insulin sensitivity. Cell Metab. (2008) 7:485–95. doi: 10.1016/j.cmet.2008.04.002 18522830 PMC2586840

[146] Bouhlel MA Derudas B Rigamonti E Dièvart R Brozek J Haulon S . PPARgamma activation primes human monocytes into alternative M2 macrophages with anti-inflammatory properties. Cell Metab. (2007) 6:137–43. doi: 10.1016/j.cmet.2007.06.010 17681149

[147] Lee YS Kim JW Osborne O Oh DY Sasik R Schenk S . Increased adipocyte O2 consumption triggers HIF-1α, causing inflammation and insulin resistance in obesity. Cell. (2014) 157:1339–52. doi: 10.1016/j.cell.2014.05.012 24906151 PMC4114226

[148] O'Rourke RW White AE Metcalf MD Olivas AS Mitra P Larison WG . Hypoxia-induced inflammatory cytokine secretion in human adipose tissue stromovascular cells. Diabetologia. (2011) 54:1480–90. doi: 10.1007/s00125-011-2103-y PMC3159546 21400042

[149] Hueso L Ortega R Selles F Wu-Xiong NY Ortega J Civera M . Upregulation of angiostatic chemokines IP-10/CXCL10 and I-TAC/CXCL11 in human obesity and their implication for adipose tissue angiogenesis. Int J Obes (Lond). (2018) 42:1406–17. doi: 10.1038/s41366-018-0102-5 29795466

[150] Martinez-Santibañez G Lumeng CN . Macrophages and the regulation of adipose tissue remodeling. Annu Rev Nutr. (2014) 34:57–76. doi: 10.1146/annurev-nutr-071812-161113 24850386

[151] Marcelin G Silveira ALM Martins LB Ferreira AV Clément K . Deciphering the cellular interplays underlying obesity-induced adipose tissue fibrosis. J Clin Invest. (2019) 129:4032–40. doi: 10.1172/jci129192 31498150 PMC6763252

[152] Wellen KE Hotamisligil GS . Obesity-induced inflammatory changes in adipose tissue. J Clin Invest. (2003) 112:1785–8. doi: 10.1172/jci20514 14679172 PMC297006

[153] Meli R Mattace Raso G Calignano A . Role of innate immune response in non-alcoholic fatty liver disease: metabolic complications and therapeutic tools. Front Immunol. (2014) 5:177. doi: 10.3389/fimmu.2014.00177 24795720 PMC4005965

[154] Gentek R Molawi K Sieweke MH . Tissue macrophage identity and self-renewal. Immunol Rev. (2014) 262:56–73. doi: 10.1111/imr.12224 25319327

[155] Kumar S Duan Q Wu R Harris EN Su Q . Pathophysiological communication between hepatocytes and non-parenchymal cells in liver injury from NAFLD to liver fibrosis. Adv Drug Delivery Rev. (2021) 176:113869. doi: 10.1016/j.addr.2021.113869 34280515 PMC11792083

[156] Guillot A Tacke F . Liver macrophages: Old dogmas and new insights. Hepatol Commun. (2019) 3:730–43. doi: 10.1002/hep4.1356 31168508 PMC6545867

[157] Hardy T Oakley F Anstee QM Day CP . Nonalcoholic fatty liver disease: Pathogenesis and disease spectrum. Annu Rev Pathol. (2016) 11:451–96. doi: 10.1146/annurev-pathol-012615-044224 26980160

[158] Morinaga H Mayoral R Heinrichsdorff J Osborn O Franck N Hah N . Characterization of distinct subpopulations of hepatic macrophages in HFD/obese mice. Diabetes. (2015) 64:1120–30. doi: 10.2337/db14-1238 25315009 PMC4375077

[159] Obstfeld AE Sugaru E Thearle M Francisco AM Gayet C Ginsberg HN . C-C chemokine receptor 2 (CCR2) regulates the hepatic recruitment of myeloid cells that promote obesity-induced hepatic steatosis. Diabetes. (2010) 59:916–25. doi: 10.2337/db09-1403 20103702 PMC2844839

[160] Krenkel O Puengel T Govaere O Abdallah AT Mossanen JC Kohlhepp M . Therapeutic inhibition of inflammatory monocyte recruitment reduces steatohepatitis and liver fibrosis. Hepatology. (2018) 67:1270–83. doi: 10.1002/hep.29544 28940700

[161] Karlmark KR Weiskirchen R Zimmermann HW Gassler N Ginhoux F Weber C . Hepatic recruitment of the inflammatory Gr1+ monocyte subset upon liver injury promotes hepatic fibrosis. Hepatology. (2009) 50:261–74. doi: 10.1002/hep.22950 19554540

[162] Tomita K Tamiya G Ando S Ohsumi K Chiyo T Mizutani A . Tumour necrosis factor alpha signalling through activation of Kupffer cells plays an essential role in liver fibrosis of non-alcoholic steatohepatitis in mice. Gut. (2006) 55:415–24. doi: 10.1136/gut.2005.071118 16174657 PMC1856073

[163] Stienstra R Saudale F Duval C Keshtkar S Groener JE van Rooijen N . Kupffer cells promote hepatic steatosis via interleukin-1beta-dependent suppression of peroxisome proliferator-activated receptor alpha activity. Hepatology. (2010) 51:511–22. doi: 10.1002/hep.23337 20054868

[164] Miura K Yang L van Rooijen N Ohnishi H Seki E . Hepatic recruitment of macrophages promotes nonalcoholic steatohepatitis through CCR2. Am J Physiol Gastrointest Liver Physiol. (2012) 302:G1310–21. doi: 10.1152/ajpgi.00365.2011 22442158 PMC3378163

[165] Han YH Kim HJ Na H Nam MW Kim JY Kim JS . RORα induces KLF4-mediated M2 polarization in the liver macrophages that protect against nonalcoholic steatohepatitis. Cell Rep. (2017) 20:124–35. doi: 10.1016/j.celrep.2017.06.017 28683306

[166] Luo W Xu Q Wang Q Wu H Hua J . Effect of modulation of PPAR-γ activity on Kupffer cells M1/M2 polarization in the development of non-alcoholic fatty liver disease. Sci Rep. (2017) 7:44612. doi: 10.1038/srep44612 28300213 PMC5353732

[167] Huang H Balzer NR Seep L Splichalova I Blank-Stein N Viola MF . Kupffer cell programming by maternal obesity triggers fatty liver disease. Nature. (2025) 644:790–8. doi: 10.1038/s41586-025-09190-w 40533564 PMC12367551

[168] MaC WangS DongB TianY . Metabolic reprogramming of immune cells in MASH. Hepatology. (2025). doi: 10.1097/hep.0000000000001371 40324062

[169] Chen P Zhu Z Chen W Xiong Z Shu K Sun M . Glycolysis drives STING signaling to promote M1-macrophage polarization and aggravate liver fibrosis. Int J Biol Sci. (2025) 21:6411–29. doi: 10.7150/ijbs.115073 41208891 PMC12594590

[170] Liu J Wu H Zhou L Jin Q Ji H Hu B . DC-SIGN(+) macrophages alleviate metabolic dysfunction-associated steatotic liver disease via fine-tuning TLR4 signaling and inflammatory secretory profiles. Metabolism. (2026) 175:156453. doi: 10.1016/j.metabol.2025.156453 41270396

[171] Wen W Liu Z Tan W Tan Y Li W Wan J . Integrating multi-omics and machine learning systematically deciphers cellular heterogeneity and fibrotic regulatory networks in the progression from MASLD to MASH. NPJ Digit Med. (2026) 9:167. doi: 10.1038/s41746-026-02352-8 41545636 PMC12913776

[172] Khomich O Ivanov AV Bartosch B . Metabolic hallmarks of hepatic stellate cells in liver fibrosis. Cells. (2019) 9:24. doi: 10.3390/cells9010024 31861818 PMC7016711

[173] Hirsova P Gores GJ . Death receptor-mediated cell death and proinflammatory signaling in nonalcoholic steatohepatitis. Cell Mol Gastroenterol Hepatol. (2015) 1:17–27. doi: 10.1016/j.jcmgh.2014.11.005 25729762 PMC4340657

[174] Pradere JP Kluwe J De Minicis S Jiao JJ Gwak GY Dapito DH . Hepatic macrophages but not dendritic cells contribute to liver fibrosis by promoting the survival of activated hepatic stellate cells in mice. Hepatology. (2013) 58:1461–73. doi: 10.1002/hep.26429 23553591 PMC3848418

[175] Canbay A Feldstein AE Higuchi H Werneburg N Grambihler A Bronk SF . Kupffer cell engulfment of apoptotic bodies stimulates death ligand and cytokine expression. Hepatology. (2003) 38:1188–98. doi: 10.1053/jhep.2003.50472 14578857

[176] Hellerbrand C Stefanovic B Giordano F Burchardt ER Brenner DA . The role of TGFbeta1 in initiating hepatic stellate cell activation *in vivo*. J Hepatol. (1999) 30:77–87. doi: 10.1016/s0168-8278(99)80010-5 9927153

[177] Ioannou GN Subramanian S Chait A Haigh WG Yeh MM Farrell GC . Cholesterol crystallization within hepatocyte lipid droplets and its role in murine NASH. J Lipid Res. (2017) 58:1067–79. doi: 10.1194/jlr.m072454 28404639 PMC5456359

[178] Ioannou GN Van Rooyen DM Savard C Haigh WG Yeh MM Teoh NC . Cholesterol-lowering drugs cause dissolution of cholesterol crystals and disperse Kupffer cell crown-like structures during resolution of NASH. J Lipid Res. (2015) 56:277–85. doi: 10.1194/jlr.m053785 25520429 PMC4306682

[179] Wallace MC Friedman SL Mann DA . Emerging and disease-specific mechanisms of hepatic stellate cell activation. Semin Liver Dis. (2015) 35:107–18. doi: 10.1055/s-0035-1550060 25974897

[180] Govaere O Petersen SK Martinez-Lopez N Wouters J Van Haele M Mancina RM . Macrophage scavenger receptor 1 mediates lipid-induced inflammation in non-alcoholic fatty liver disease. J Hepatol. (2022) 76:1001–12. doi: 10.1016/s0168-8278(20)30598-5 PMC7619241 34942286

[181] Su T He Y Wang M Zhou H Huang Y Ye M . Macrophage-hepatocyte circuits mediated by grancalcin aggravate the progression of metabolic dysfunction associated steatohepatitis. Adv Sci (Weinh). (2024) 11:e2406500. doi: 10.1002/advs.202406500 39279458 PMC11558151

[182] Kuo HR Liu KY Clair Chiou HY Chung YF Wang LT Wang CW . Macrophage MRC2 deficiency mitigates HFD-induced MASLD by downregulating CD147-regulated TNF-α production. JHEP Rep. (2025) 7:101601. doi: 10.1016/j.jhepr.2025.101601 41362709 PMC12682121

[183] Eguchi K Manabe I . Macrophages and islet inflammation in type 2 diabetes. Diabetes Obes Metab. (2013) 15:152–8. doi: 10.1111/dom.12168 24003932

[184] Ehses JA Perren A Eppler E Ribaux P Pospisilik JA Maor-Cahn R . Increased number of islet-associated macrophages in type 2 diabetes. Diabetes. (2007) 56:2356–64. doi: 10.2337/db06-1650 17579207

[185] Cucak H Grunnet LG Rosendahl A . Accumulation of M1-like macrophages in type 2 diabetic islets is followed by a systemic shift in macrophage polarization. J Leukoc Biol. (2014) 95:149–60. doi: 10.1189/jlb.0213075 24009176

[186] Ying W Fu W Lee YS Olefsky JM . The role of macrophages in obesity-associated islet inflammation and β-cell abnormalities. Nat Rev Endocrinol. (2020) 16:81–90. doi: 10.1038/s41574-019-0286-3 31836875 PMC8315273

[187] Donath MY Dalmas É Sauter NS Böni-Schnetzler M . Inflammation in obesity and diabetes: islet dysfunction and therapeutic opportunity. Cell Metab. (2013) 17:860–72. doi: 10.1016/j.cmet.2013.05.001 23747245

[188] Calderon B Carrero JA Ferris ST Sojka DK Moore L Epelman S . The pancreas anatomy conditions the origin and properties of resident macrophages. J Exp Med. (2015) 212:1497–512. doi: 10.1084/jem.20150496 26347472 PMC4577842

[189] Igoillo-Esteve M Marselli L Cunha DA Ladrière L Ortis F Grieco FA . Palmitate induces a pro-inflammatory response in human pancreatic islets that mimics CCL2 expression by beta cells in type 2 diabetes. Diabetologia. (2010) 53:1395–405. doi: 10.1007/s00125-010-1707-y 20369226

[190] Dasu MR Jialal I . Free fatty acids in the presence of high glucose amplify monocyte inflammation via Toll-like receptors. Am J Physiol Endocrinol Metab. (2011) 300:E145–54. doi: 10.1152/ajpendo.00490.2010 20959532 PMC3023203

[191] Maedler K Sergeev P Ris F Oberholzer J Joller-Jemelka HI Spinas GA . Glucose-induced beta cell production of IL-1beta contributes to glucotoxicity in human pancreatic islets. J Clin Invest. (2002) 110:851–61. doi: 10.1172/jci200215318 12235117 PMC151125

[192] Eguchi K Manabe I Oishi-Tanaka Y Ohsugi M Kono N Ogata F . Saturated fatty acid and TLR signaling link β cell dysfunction and islet inflammation. Cell Metab. (2012) 15:518–33. doi: 10.1016/j.cmet.2012.01.023 22465073

[193] Ward MG Li G Hao M . Apoptotic β-cells induce macrophage reprogramming under diabetic conditions. J Biol Chem. (2018) 293:16160–73. doi: 10.1074/jbc.ra118.004565 30213857 PMC6200952

[194] Park YJ Warnock GL Ao Z Safikhan N Meloche M Asadi A . Dual role of interleukin-1β in islet amyloid formation and its β-cell toxicity: Implications for type 2 diabetes and islet transplantation. Diabetes Obes Metab. (2017) 19:682–94. doi: 10.1007/978-981-16-4597-6_24 28058779

[195] Westwell-Roper CY Ehses JA Verchere CB . Resident macrophages mediate islet amyloid polypeptide-induced islet IL-1β production and β-cell dysfunction. Diabetes. (2014) 63:1698–711. doi: 10.2337/db13-0863 24222351

[196] Chen X Shao J Brandenburger I Qian W Hahnefeld L Bonnavion R . FFAR4-mediated IL-6 release from islet macrophages promotes insulin secretion and is compromised in type-2 diabetes. Nat Commun. (2025) 16:3422. doi: 10.1038/s41467-025-58706-5 40210633 PMC11986018

[197] de Souza CO Paschoal VA Sun X Vishvanath L Zhang Q Shao M . GPR92 activation in islet macrophages controls β cell function in a diet-induced obesity model. J Clin Invest. (2022) 132:e160097. doi: 10.1172/jci160097 36066975 PMC9621135

[198] Sokolova M Sahraoui A Høyem M Øgaard J Lien E Aukrust P . NLRP3 inflammasome mediates oxidative stress-induced pancreatic islet dysfunction. Am J Physiol Endocrinol Metab. (2018) 315:E912–23. doi: 10.1152/ajpendo.00461.2017 30016155

[199] Legrand-Poels S Esser N L'homme L Scheen A Paquot N Piette J . Free fatty acids as modulators of the NLRP3 inflammasome in obesity/type 2 diabetes. Biochem Pharmacol. (2014) 92:131–41. doi: 10.1016/j.bcp.2014.08.013 25175736

[200] Wang C Geng B Cui Q Guan Y Yang J . Intracellular and extracellular adenosine triphosphate in regulation of insulin secretion from pancreatic β cells (β). J Diabetes. (2014) 6:113–19. doi: 10.1111/1753-0407.12098 24134160

[201] Weitz JR Makhmutova M Almaça J Stertmann J Aamodt K Brissova M . Mouse pancreatic islet macrophages use locally released ATP to monitor beta cell activity. Diabetologia. (2018) 61:182–92. doi: 10.1007/s00125-017-4416-y 28884198 PMC5868749

[202] Hong Z Chen S Sun J Cheng D Guo H Mei J . STING signaling in islet macrophages impairs insulin secretion in obesity. Sci China Life Sci. (2024) 67:345–59. doi: 10.1007/s11427-022-2371-9 37906411

[203] Brissova M Aamodt K Brahmachary P Prasad N Hong JY Dai C . Islet microenvironment, modulated by vascular endothelial growth factor-A signaling, promotes β cell regeneration. Cell Metab. (2014) 19:498–511. doi: 10.1016/j.cmet.2015.09.014 24561261 PMC4012856

[204] Xiao X Gaffar I Guo P Wiersch J Fischbach S Peirish L . M2 macrophages promote beta-cell proliferation by up-regulation of SMAD7. Proc Natl Acad Sci USA. (2014) 111:E1211–20. doi: 10.1073/pnas.1321347111 24639504 PMC3977272

[205] Criscimanna A Coudriet GM Gittes GK Piganelli JD Esni F . Activated macrophages create lineage-specific microenvironments for pancreatic acinar- and β-cell regeneration in mice. Gastroenterology. (2014) 147:1106–1118.e11. doi: 10.1053/j.gastro.2014.08.008 25128759

[206] Grosjean A Jalon A Leveau C Diedisheim M Bejarano DA Cuenco J . An islet-resident macrophage antioxidant program preserves β cell physiology. Sci Immunol. (2025) 10:eadz5181. doi: 10.1126/sciimmunol.adz5181 41237222

[207] Zhang S Song C Zhang W . Jinkui Shenqi pills improve pancreatic islet function in diabetes by modulating macrophage inflammation through the succinate/HIF1α axis. Phytomedicine. (2025) 148:157043. doi: 10.1016/j.phymed.2025.157043 41022007

[208] Qiu Z Shang D . The role of pancreatic islet macrophages in type 2 diabetes mellitus: from underlying pathological mechanisms to therapeutic target discovery. Metabol Open. (2025) 28:100418. doi: 10.1016/j.metop.2025.100418 41321403 PMC12664484

[209] Mou L Wang TB Lu Y Wu Z Chen Y Luo Z . Targeting macrophages and ion homeostasis in T2D: new genes and therapeutic pathways identified. Front Immunol. (2025) 16:1514243. doi: 10.3389/fimmu.2025.1514243 40895573 PMC12391075

[210] Zhou Q Li M Zhang J Zhou X Zhu Q Ni H . PPARγ activation reduces pancreatic beta cell death in type 1 diabetes by decreasing heparanase-dependent insulitis. Int Immunopharmacol. (2025) 162:115183. doi: 10.1016/j.intimp.2025.115183 40628043

[211] Zhang Y Cong R Lv T Liu K Chang X Li Y . Islet-resident macrophage-derived miR-155 promotes β cell decompensation via targeting PDX1. iScience. (2024) 27:109540. doi: 10.1016/j.isci.2024.109540 38577099 PMC10993184

[212] Qian B Yang Y Tang N Wang J Sun P Yang N . M1 macrophage-derived exosomes impair beta cell insulin secretion via miR-212-5p by targeting SIRT2 and inhibiting Akt/GSK-3β/β-catenin pathway in mice. Diabetologia. (2021) 64:2037–51. doi: 10.1007/s00125-021-05489-1 34117507

[213] Yamashita YM Inaba M Buszczak M . Specialized intercellular communications via cytonemes and nanotubes. Annu Rev Cell Dev Biol. (2018) 34:59–84. doi: 10.1146/annurev-cellbio-100617-062932 30074816 PMC6404750

[214] Alrahimi JS Ahmed FA Haneef AA Atar D . Integrative perspectives on atherosclerosis: From molecular mechanisms to therapeutic approaches. Saudi J Med Med Sci. (2025) 13:239–52. doi: 10.4103/sjmms.sjmms_18_25 41163826 PMC12560991

[215] Shu B Chen X Liu Z Tang H Yang B Fu C . Pleiotropic effects of SGLT2 inhibitors: A focus on macrophage-mediated action. Pharmacol Res. (2025) 222:108046. doi: 10.1016/j.phrs.2025.108046 41285328

[216] Chen G Yu Y Zhu Y Nagashimada M Wang Y Nagata N . Cenicriviroc suppresses and reverses steatohepatitis by regulating macrophage infiltration and M2 polarization in mice. Endocrinology. (2024) 165:bqae069. doi: 10.1210/endocr/bqae069 38862137

[217] Ryu S Spadaro O Sidorov S Lee AH Caprio S Morrison C . Reduction of SPARC protects mice against NLRP3 inflammasome activation and obesity. J Clin Invest. (2023) 133:e169173. doi: 10.1172/jci169173 37781916 PMC10541189

[218] Xiao Q Wang C Yang J Gao X Chen Z Niu Z . Dual-targeting nanoplatform to regulate metabolic disorders: From obesity to obesity-related metabolic dysfunction-associated steatotic liver disease. Nano Lett. (2025) 25:15416–27. doi: 10.1021/acs.nanolett.5c04561 41062304

[219] Kim J Sinha S Solomon M Perez-Herrero E Hsu J Tsinas Z . Co-coating of receptor-targeted drug nanocarriers with anti-phagocytic moieties enhances specific tissue uptake versus non-specific phagocytic clearance. Biomaterials. (2017) 147:14–25. doi: 10.1016/j.biomaterials.2017.08.045 28923682 PMC5667353

[220] Lao A Li W Sun Y Cao Y Zhuang Y Wu J . Metabolic and immunomodulatory control of type 2 diabetes via generating cellular itaconate reservoirs by inflammatory-targeting gene-therapy nanovesicles. Trends Biotechnol. (2026) 44:170–92. doi: 10.1016/j.tibtech.2025.07.025 40925799

